# Dark Sweet Cherry Anthocyanins Suppressed Triple-Negative Breast Cancer Pulmonary Metastasis and Downregulated Genes Associated with Metastasis and Therapy Resistance *In Vivo*

**DOI:** 10.3390/ijms26157225

**Published:** 2025-07-25

**Authors:** Ana Nava-Ochoa, Lauren W. Stranahan, Rodrigo San-Cristobal, Susanne U. Mertens-Talcott, Giuliana D. Noratto

**Affiliations:** 1 Department of Food Science and Technology, Texas A & M University, College Station, TX 77843, USA; ana.nava@tamu.edu (A.N.-O.); r.sancristobalblanco@ag.tamu.edu (R.S.-C.); smtalcott@tamu.edu (S.U.M.-T.); 2 Department of Veterinary Pathobiology, School of Veterinary Medicine & Biomedical Sciences, Texas A & M University, College Station, TX 77843, USA; lstranahan@cvm.tamu.edu

**Keywords:** triple negative breast cancer, dark sweet cherries, anthocyanins, metastasis

## Abstract

Dark sweet cherries (DSC) phytochemicals have emerged as a promising dietary strategy to combat triple-negative breast cancer (TNBC). This study explored the effects of DSC extract rich in anthocyanins (ACN) as a chemopreventive agent and as a complement to doxorubicin (DOX) in treating TNBC tumors and metastasis using a 4T1 syngeneic animal model. Initiating ACN intake as a chemopreventive one week before 4T1 cell implantation significantly delayed tumor growth without any signs of toxicity. Both DOX treatment and the combination of DOX-ACN effectively delayed tumor growth rate, but DOX-ACN allowed for body weight gain, which was hindered by DOX alone. As a chemopreventive, ACN downregulated metastasis- and immune-suppression-related genes, including STAT3, Snail1, mTOR, SIRT1, TGFβ1, IKKβ, and those unaffected by DOX alone, such as HIF, Cd44, and Rgcc32. Correlations between mRNA levels seen in control and DOX groups were absent in ACN and/or DOX-ACN groups, indicating that Cd44, mTOR, Rgcc32, SIRT1, Snail1, and TGFβ1 may be ACN targets. The DOX-ACN treatment showed a trend toward enhanced efficacy involving CREB, PI3K, Akt-1, and Vim compared to DOX alone. Particularly, ACN significantly suppressed lung metastasis compared to the other groups. ACN also decreased the frequency and incidence of metastasis in the liver, heart, kidneys, and spleen, while their metastatic area (%) and number of breast cancer (BC) metastatic tumor nodules were lowered without reaching significance. Further research is needed to explore the efficacy of combining ACN with drug therapy in the context of drug resistance.

## 1. Introduction

According to the World Health Organization, breast cancer (BC) caused 670,000 deaths globally and was the most common cancer in women in 157 countries out of 185 in 2022 [1]. BC is the most common malignancy among women worldwide, with triple-negative breast cancer (TNBC) being the most aggressive and carrying the worst prognosis of all BC subtypes. The TNBC subtype lacks estrogen receptor (ER), progesterone receptor (PR), and human epidermal growth factor receptor 2 (HER2) expression, which makes conventional hormonal therapy ineffective. Furthermore, there are no clinically approved therapies specifically targeting TNBC. Most chemotherapeutic drugs focus on single-target mechanisms, and TNBC has a heterogeneous biology. Therefore, at advanced cancer stages, their clinical efficacy is limited by side effects and a tendency to develop chemoresistance over time.

T lymphocytes, which express the CD3+ complex on their surface, and natural killer (NK) cells have important roles in antitumor immunity and suppression of tumor-associated inflammation. The evaluation of cell counts and functionality of CD3+ have increasingly become the research of interest in cancer immunology [2]. These cells work in concert to recognize and kill tumor cells. Patients with low levels of cytotoxic T cells and NK cell activity are predisposed to higher tumor progression. On the contrary, their presence has been associated with better outcome, mainly for patients with TNBC, whose tumors are infiltrated by T cells more often than the hormone receptor-positive tumors [3].

Metastasis occurs when BC cells break away from the primary tumor and travel through the bloodstream or lymphatic system to form new (secondary) tumors in different organs or tissues. Among the most common sites of metastasis are the bones, lungs, liver, and brain. The mortality of BC patients is largely caused by the spread of cancer cells to organs, leading to organotropic metastasis of tumors.

Cancer drug development is expensive and may not promise efficacy without side effects. Dietary phytochemicals may improve the efficacy of drugs as phyto-adjuvants with no side effects, helping in the suppression of BC progression and combating drug-resistance [4]. Therefore, combining drugs with noncytotoxic dietary phytochemicals that have complementary mechanisms of action may enhance chemotherapy outcomes, help to overcome chemoresistance, and reduce the need for increasing drug dosage and associated toxicity.

The intake of dietary phytochemicals, e.g., anthocyanins, has been shown to be effective as a preventive strategy to disrupt cancer in its early stage, thus offering a proactive approach to reducing cancer risk. This strategy, called chemoprevention, seeks to lower the risk of cancer in individuals who may be at increased risk due to genetic factors, lifestyle habits, environmental exposures, or other predispositions. Additionally, interventions in patients with premalignant lesions that could develop into invasive disease are referred to as secondary chemoprevention. Phytochemicals have been demonstrated to be effective in primary and secondary chemoprevention by targeting multiple pathways involved in cancer development and progression, as well as in preventing recurrence in individuals who have been treated for cancer or have precancerous conditions [5].

Among the potential targets identified to hinder TNBC cell growth, invasion and metastasis are mitogen activated protein kinases (MAPK), and phosphoinositide 3-kinase (PI3K)/protein kinase B (AKT)/mechanistic target of rapamycin (mTOR) signaling pathways [6].

DSC extract rich in anthocyanins (ACN) and its main components cyanidin-3-O-rutinoside (1103.65 ± 167.09 mg/L DSC concentrated juice), cyanidin-3-glucoside (123.36 ± 58.53 mg/L DSC concentrated juice), peonidin-3-O-rutinoside (113.30 ± 70.63 mg/L DSC concentrated juice), and other unidentified (49.80 ± 40.95 mg/L DSC concentrated juice), accounting for a total of 1390.12 ± 261.38 mg/L DSC concentrated juice [7], were shown to suppress oxidative stress, proliferation, and angiogenesis in the 4T1 TNBC cells through the modulation of PI3K/AKT/mTOR pathway and the MAPKs/extracellular signal-regulated kinases (ERK)/cAMP response element-binding (CREB) axis [8].

The PI3K/AKT/mTOR signaling pathway has been shown to play a role in promoting epithelial-to-mesenchymal transition (EMT), which is the conversion of polarized epithelial cells into non-polarized mesenchymal cells, leading to invasion and motility. It does so by regulating key signaling molecules and transcription factors involved in EMT, such as zinc finger 1 (Snail1), Twist, and Zeb1/2. Therefore, the EMT process leading to increased invasiveness, metastatic potential, and resistance to therapy might be prevented and/or slowed down by inhibiting the PI3K/Akt/mTOR pathway [9].

Considering that metastatic cancer is often lethal, with significantly lower survival rates at this advanced stage, the exploration of phytochemicals for the prevention and/or treatment of metastasis is crucial. However, studies on the anti-metastatic activity of phytochemicals have lagged, especially when compared to studies focusing on cancer initiation and progression stages.

Results from a preliminary study with the mouse syngeneic model orthotopically implanted 4T1 BC cells [8] showed that total angiogenesis area in the lungs, the most common site for TNBC metastasis, was significantly reduced in mice fed ACN compared to controls, as were mRNA levels of Centromere Protein F (Cenpf), which has been associated with lung metastasis [10]. These preliminary results strongly suggested that ACN may protect against lung metastasis.

The goal of this study was to investigate the effects of ACN as a chemopreventive dietary agent and as phyto-adjuvant or complementary to the chemotherapeutic drug DOX on modulation of gene expression in BC tumors and metastasis in distant organs using the syngeneic animal model orthotopically implanted 4T1 BC cells, which mimic stage IV human BC.

## 2. Results

### 2.1. Body Weight (BW) and Tumor Growth Rate

**Figure 1:**
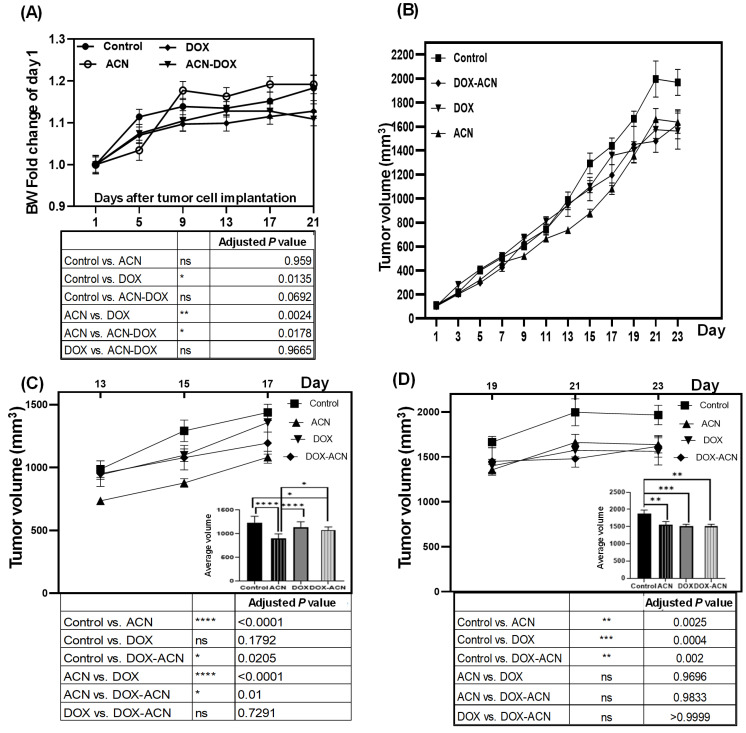
ACN as chemopreventive and as phyto-adjuvant to DOX treatment supported body weight (BW) gain, while it delayed tumor growth. (**A**) BW fold increase of day 1. (**B**) Tumor volume between days 1 and 23. (**C**) Tumor volume between days 13 and 17. (**D**) Tumor volume between days 19 and 23. Scatter plots display tumor volume over time, while the bar graphs present the average tumor volume with statistical significance indicated. Day 1 was set when the tumor volume reached 100 mm^3^. Data from BW and tumor volume are mean and SEM (upper panels). Data was analyzed by 2-way ANOVA, followed by Tukey’s multiple comparisons test (lower panels) (ns, *p* > 0.05), (*, *p* ≤ 0.05), (**, *p* ≤ 0.01), (***, *p* ≤ 0.001), (****, *p* ≤ 0.0001).

Results from BW changes, assessed as fold increase of day 1 (4T1 tumor cell implantation day), showed to be significant between experimental groups (Figure 1A). DOX treatment impeded BW increase compared to both the control and ACN treatments. Similarly, the DOX-ACN group exhibited hindered BW increase compared to the ACN group.

**Figure 2:**
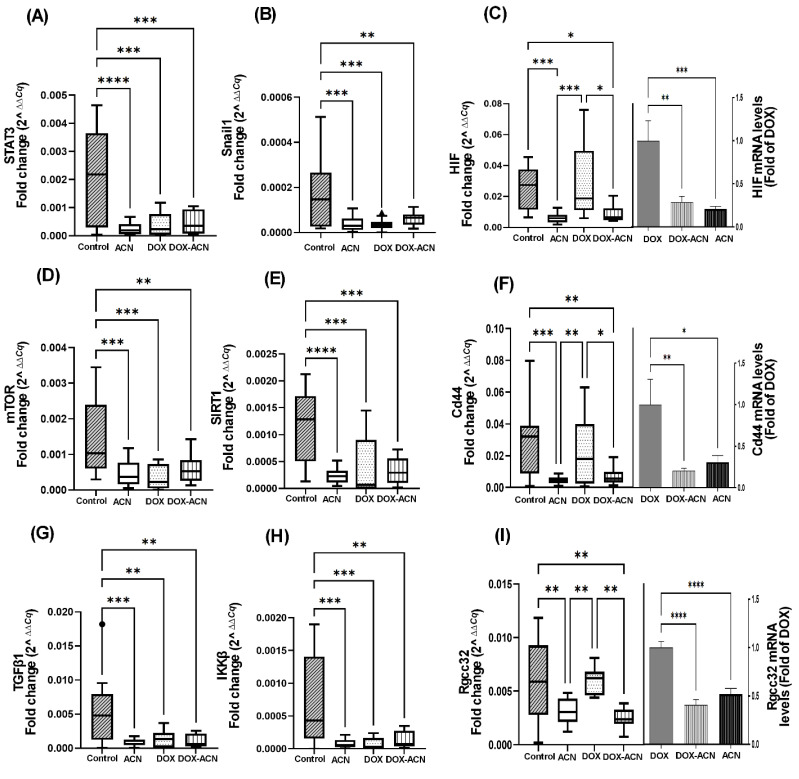
ACN as chemopreventive and as a complement to DOX treatment downregulated the expression of genes associated with BC metastasis. (**A**) STAT3 mRNA levels. (**B**) Snail1 mRNA levels. (**C**) HIF mRNA levels (left panel) and in comparison to DOX (right panel). (**D**) mTOR mRNA levels. (**E**) SIRT1 mRNA levels. (**F**) Cd44 mRNA levels and in comparison to DOX (right panel). (**G**) TGFβ1 mRNA levels. (**H**) IKKβ mRNA levels. (**I**) Rgcc32 mRNA levels. (left panel) and in comparison to DOX (right panel). Data from left panels are fold change (2^^∆∆C*q*^) values. Box-plot central box spans from the first quartile (Q1) to the third quartile (Q3). The line inside the box represents the median (Q2). The whiskers extend from the box to the smallest and largest values within 1.5× interquartile range (IQR) of the lower and upper quartiles. Data from fold of DOX are mean and SEM relative to DOX values. Analysis was performed using one-way ANOVA, followed by uncorrected Fisher’s LSD test, (*, *p* ≤ 0.05), (**, *p* ≤ 0.01), (***, *p* ≤ 0.001), (****, *p* ≤ 0.0001).

Tumor growth rate was analyzed only until day 23 because it had the highest number of experimental units. Results showed no difference in tumor growth rate between experimental groups within days 1–11 (Figure 1B), and there was no difference in the weight of excised tumors at the end of the study between groups. However, significant differences in tumor growth rate were observed during days 13–23 in this study (Figure 1C and Figure 2D).

Interestingly, there was very high statistical significance for control vs. ACN (****, *p* ≤ 0.001), and for ACN vs. DOX (****) (Figure 1C). Furthermore, within days 13–17, treatment with DOX alone did not significantly suppress tumor growth compared to the control group, whereas the DOX-ACN combination showed a noticeable inhibitory effect (*) (Figure 1C). The averages tumor volumes by intervention group, within days 13–17 were as follows: control group—1365.85 mm^3^; ACN group—979.73 mm^3^ (28% reduction compared to control); DOX group—1228 mm^3^ (10% reduction); and DOX-ACN group—1137 mm^3^ (16.7% reduction) (Figure 1C). In general, ACN group differs from everyone. It yields much lower mean values than control (–341 units) and the two DOX-containing groups (–235 units vs. DOX, –177 units vs. DOX-ACN).

For days 19–23, ACN (**), and both DOX alone (*) and the DOX-ACN combination (**), significantly suppressed tumor growth compared to the control group (Figure 1D). The average tumor volumes were as follows: control group—1982 mm^3^; ACN group—1649 mm^3^ (16% reduction vs. control); DOX group—1568 mm^3^ (20% reduction); and DOX-ACN group—1548 mm^3^ (22% reduction) (Figure 1D).

### 2.2. Tumor Tissue Analyses

Analyses were performed only in animals terminated between days 17 and 23 to analyze the highest number of experimental units and to decrease the influence of time on metastasis progress.

#### Gene Expression

Results from genes that were significantly modulated in experimental groups are shown in Supplementary Table S1, and for those that were not significantly modulated in Supplementary Table S2.

STAT3 mRNA levels were significantly downregulated in the ACN, DOX, and DOX-ACN groups, reaching 0.12-, 0.18-, and 0.23-fold of the control levels, respectively (Figure 2A and Supplementary Table S1). The treatments also led to significant downregulation of Snail1 and other inflammation-related genes, including TGFβ1 and IKKβ (Figure 2B,G,H). In contrast, the expression levels of STAT3 downstream target genes—such as VEGFA, IL-6, MMP13, Bcl2, and Twist1—remained comparable across all experimental groups (Supplementary Table S2), likely due to tumor excision occurring at a stage when metastasis was already underway. Snail1 gene expression followed a pattern like STAT3, showing downregulation in the ACN, DOX, and DOX-ACN groups to 0.23-, 0.23-, and 0.36-fold of the control levels, respectively (Figure 2B and Supplementary Table S1).

HIF gene expression was significantly downregulated only in the ACN group (0.24-fold compared to the control, *p* = 0.0029) and reduced in the DOX-ACN group (0.34-fold of the control), although the latter did not reach statistical significance due to high inter-animal variability (Figure 2C, left panel). Notably, compared to the DOX group, HIF expression was also significantly downregulated in the ACN group (0.24-fold, *p* = 0.0017) and lowered in the DOX-ACN group (0.34-fold), again without reaching significance (Figure 2C, right panel).

As a chemopreventive agent, ACN also downregulated the expression of additional STAT3-associated genes, including mTOR, SIRT1, and Cd44 (Figure 2D–F and Supplementary Table S1). mTOR expression was reduced to 0.32-, 0.25-, and 0.41-fold of the control in the ACN, DOX, and DOX-ACN groups, respectively (Figure 2D). Similarly, SIRT1 mRNA levels were suppressed to 0.21-, 0.54-, and 0.29-fold of the control by ACN, DOX, and DOX-ACN treatments, respectively (Figure 2E).

SIRTs influence EMT, in part through TGFβ signaling [11]. Consistent with previous findings, TGFβ1 mRNA levels were downregulated in the ACN, DOX, and DOX-ACN groups to 0.14-, 0.25-, and 0.21-fold of the control, respectively (Figure 2G). Similarly, IKKβ expression was significantly reduced in both the ACN and DOX groups (*p* < 0.05), reaching 0.12-fold of the control in each case (Figure 2H and Supplementary Table S1).

The results showed that Cd44 mRNA levels were reduced to 0.16- and 0.23-fold of the control in the ACN and DOX-ACN groups, respectively (Figure 2F, left panel). Notably, DOX alone did not suppress Cd44 expression; however, the DOX-ACN combination lowered Cd44 mRNA to 0.30-fold of DOX levels—an effect likely driven by ACN, as Cd44 expression in the ACN group alone was reduced to 0.20-fold of DOX (Figure 2F, right panel).

Rgcc32 expression was reduced to 0.53- and 0.42-fold of the control in the ACN and DOX-ACN groups, respectively (Figure 2I, left panel). Notably, DOX alone had no effect on Rgcc32 expression, while ACN lowered its mRNA levels to 0.52-fold of the DOX group. The combination treatment (DOX-ACN) further decreased Rgcc32 expression to 0.41-fold of the DOX group, similar to levels observed with ACN alone (Figure 2I, right panel).

**Figure 3:**
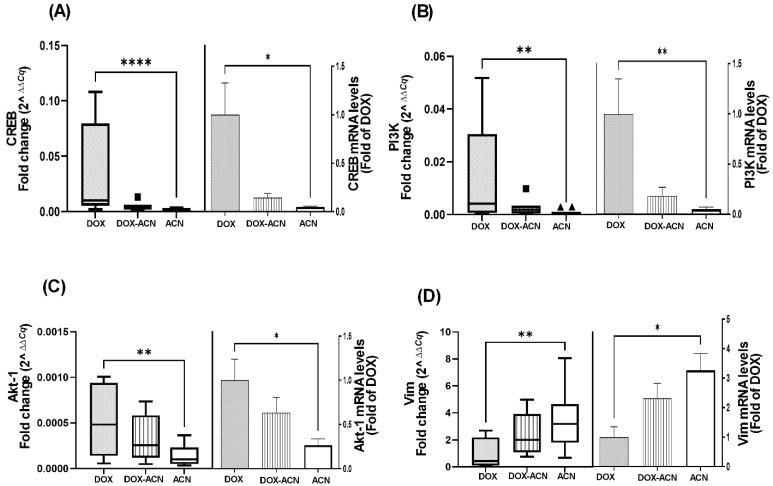
ACN intake as a chemopreventive and as adjuvant to DOX treatment downregulated the expression of genes that DOX treatment alone could not suppress. (**A**) CREB mRNA levels (left panel) and in comparison to DOX (right panel). (**B**) PI3K mRNA levels (left panel) and in comparison to DOX (right panel). (**C**) Akt-1 mRNA levels (left panel) and in comparison to DOX (right panel). (**D**) Vim mRNA levels (left panel) and in comparison to DOX (right panel). Data from left panels are fold change (2^^∆∆C*q*^) values. Box-plot central box spans from the first quartile (Q1) to the third quartile (Q3). The line inside the box represents the median (Q2). The whiskers extend from the box to the smallest and largest values within 1.5× interquartile range (IQR) of the lower and upper quartiles. Data from fold of DOX are mean and SEM relative to DOX values. Analysis was performed using the Kruskal–Wallis test followed by uncorrected Dunn’s test, (*, *p* ≤ 0.05), (**, *p* ≤ 0.01), (****, *p* ≤ 0.0001). The squares and the triangles are individual values outside 1.5×IQR.

Among the genes significantly modulated compared to the DOX group, the transcription factor CREB was markedly downregulated in the ACN group (0.05-fold of DOX, *p* = 0.0002) and reduced in the DOX-ACN group (0.15-fold of DOX), although the latter did not reach statistical significance due to high inter-animal variability (Figure 3A). Similarly, PI3K and Akt-1 mRNA levels followed the same trend, showing suppression in the ACN group (0.05- and 0.26-fold of DOX, respectively) and reduced expression in the DOX-ACN group (0.18- and 0.63-fold of DOX, respectively), without reaching statistical significance (Figure 3B,C).

Vim, a mesenchymal marker upregulated during EMT and associated with increased cell motility and metastasis, was unexpectedly elevated in the ACN and DOX-ACN groups compared to DOX alone, reaching 3.3- and 2.3-fold of DOX levels, respectively (Figure 3D). In contrast, Tjp1 mRNA levels were significantly downregulated by ACN, DOX, and DOX-ACN treatments relative to the control, with levels reduced to 0.19-, 0.35-, and 0.29-fold, respectively (Supplementary Table S1 and Supplementary Figure S1).

**Figure 4:**
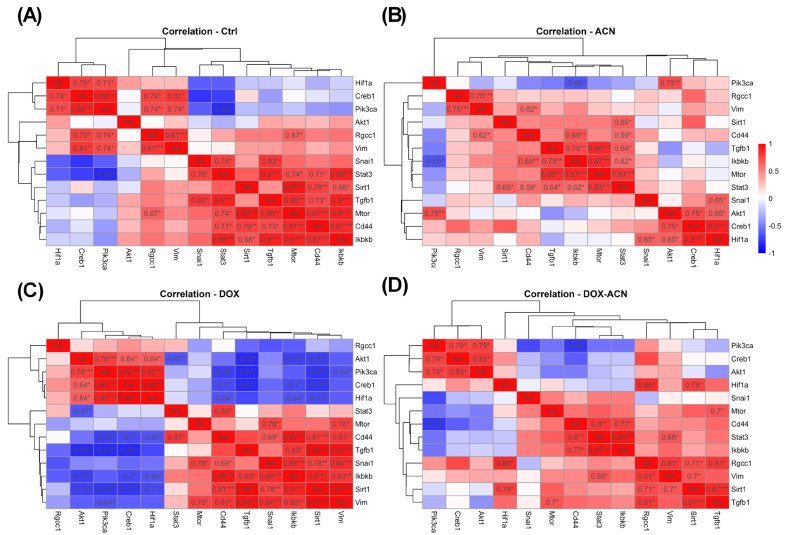
Heatmap of Spearman correlations for gene expression by treatment. (**A**) Gene expression correlations among the control group. (**B**) Gene expression correlations among the ACN group. (**C**) Gene expression correlations among the DOX group. (**D**) Gene expression correlations among the DOX-ACN group. Heatmaps display Spearman correlation coefficients between the expression levels of selected genes across four experimental groups: Ctrl (Control), ACN, DOX, and DOX-ACN. Red and blue colors indicate positive and negative correlations, respectively, with intensity proportional to the correlation strength (scale: −1 to +1). Asterisks indicate levels of statistical significance (* *p* < 0.05, ** *p* < 0.01, *** *p* < 0.001), and NA denotes missing or non-calculable correlations.

Interestingly, significant gene expression correlations observed in the control (Figure 4A) and DOX groups (Figure 4C) groups among Cd44, mTOR, Rgcc32, SIRT1, Snail1, and TGFβ1 were no longer present in the ACN (Figure 4B) or DOX-ACN (Figure 4D) groups. Similarly, in the DOX group, CREB, PI3K, Akt-1, and Vim each showed significant correlations with seven other genes (Figure 4C), whereas in the DOX-ACN group, each was correlated with only two (Figure 4D). Additionally, TGFβ1 expression was correlated with six genes in the DOX group but only three in the DOX-ACN group. IKKβ showed correlations with eight genes in the DOX group, compared to just two in the DOX-ACN group.

**Figure 5:**
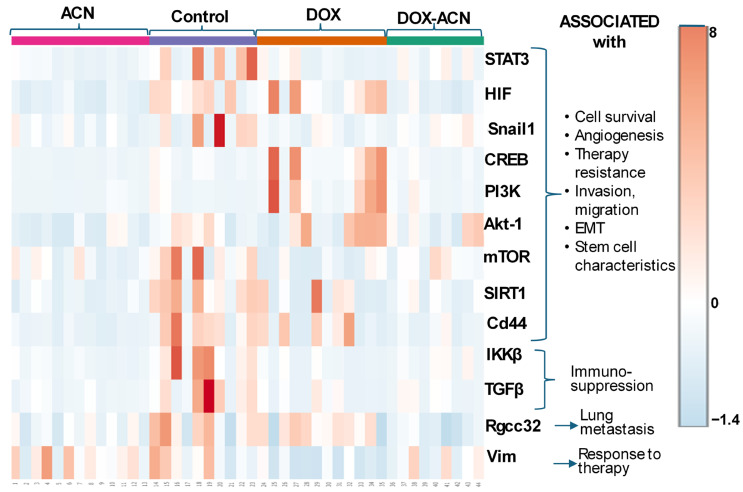
Heatmap of gene expression levels: orange indicates high levels of mRNA (gene expression), while white represents absence of mRNA or lack of gene expression. Light blue signifies downregulation of the gene, with darker shades of blue indicating greater levels of downregulation or suppression. Heatmap generated with MetaboAnalyst 6.0.

The heatmap in Figure 5 demonstrates that ACN treatment effectively decreased the expression of genes involved in cell survival, angiogenesis, therapy resistance, invasion and migration, EMT, and stem cell characteristics. Moreover, both ACN and DOX-ACN treatments downregulated genes that were not suppressed by DOX alone (Figure 5).

### 2.3. Tumor Histopathology

**Figure 6:**
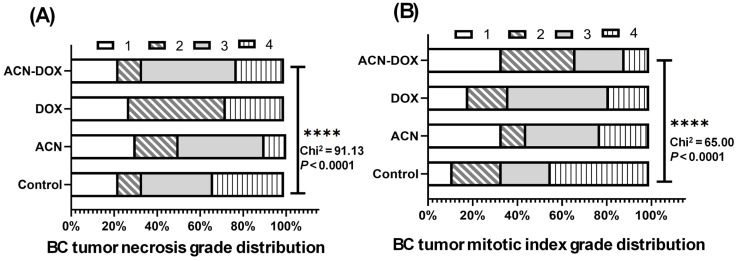
BC Necrotic grade and mitotic index grade distributions among experimental groups. (**A**) Tumor necrosis grade distribution. (**B**) Mitotic index grade distribution. Data for necrotic and mitotic index grades distribution were evaluated using contingency tables of frequency distributions and analyzed with the chi-square test. Grade 1 corresponds to area values from minimum to < 25% percentile, grade 2 from 25% percentile to < median, grade 3 from median to <75% percentile, and grade 4 from 75% percentile to maximum value, (****, *p* ≤ 0.0001).

Histopathological analysis of tumor tissues revealed necrotic areas—regions where cells died primarily due to insufficient blood supply. The extent of necrosis showed no significant differences between treatment groups (Supplementary Figure S2A). However, the distribution of necrosis grades differed significantly among groups (*p* < 0.0001), with the ACN group exhibiting the lowest percentage of grade 4 necrosis (10%) compared to the other groups (Figure 6A and Supplementary Table S3).

The mitotic index in primary tumors was comparable across groups (Supplementary Figure S2B, and Supplementary Table S3), yet the distribution of mitotic index grades varied, with the control group having the highest proportion of grade 4 (44%) (Figure 6B and Supplementary Table S3).

### 2.4. Metastasis

#### 2.4.1. Pulmonary Metastasis

**Figure 7:**
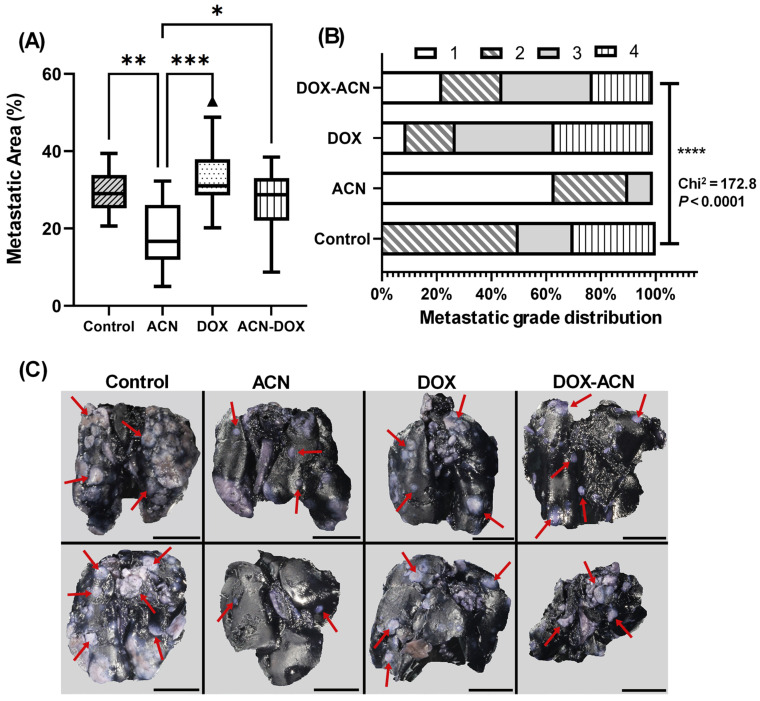
ACN intake as a chemopreventive treatment and as phyto-adjuvant alongside DOX treatment prevented the progression of lung metastasis. (**A**) Metastatic area (%). Area of white tumor nodules measured using ImageJ 1.54p. Box-plot central box spans from the first quartile (Q1) to the third quartile (Q3). The line inside the box represents the median (Q2). The whiskers extend from the box to the smallest and largest values within 1.5× interquartile range (IQR) of the lower and upper quartiles. (**B**) Metastatic grade distribution. Data from metastatic areas was transformed to quantify metastatic grade. Metastatic grade 1 corresponds to values from minimum value to < 25% percentile, grade 2 from 25% percentile to < median, grade 3 from median to < 75% percentile, and grade 4 from 75% percentile to maximum value. (**C**) Representative pictures of lungs. Inflation of lungs with India ink conducted as detailed in material and methods. The metastatic nodules indicated by arrows are clearly visible on both sides of the organ against the blue lung tissue background. Pictures were taken with a Canon EOS 80D camera with EF-S 60 mm Macro lens. The scale bar represents 1 cm. Data from metastatic area was analyzed by one-way ANOVA, followed by uncorrected Fisher’s LSD. Data from metastatic grade distribution was evaluated using contingency tables and analyzed with the chi-square test, (*, *p* ≤ 0.05), (**, *p* ≤ 0.01), (***, *p* ≤ 0.001), (****, *p* ≤ 0.0001).

Data for lung metastatic grade and percentage of metastatic area were strongly correlated (r = 0.921, ***). Analysis of metastatic areas showed that lung metastasis was significantly inhibited in the ACN group compared to the control (***). Furthermore, ACN treatment notably reduced lung metastasis relative to both the DOX (***) and DOX-ACN (*) groups. While DOX alone did not suppress lung metastasis, the DOX-ACN combination reduced metastasis, though not significantly, compared to control and DOX groups (Figure 7A). Similarly, metastatic grade distribution differed significantly among groups, with the ACN group exhibiting the highest proportion of grade 1 metastasis (63%) and no cases of grade 4 (Figure 7B and Supplementary Table S4). The results were supported by representative images showing BC metastatic nodules in the lungs (white areas) against the blue lung tissue background (Figure 7C).

#### 2.4.2. Hepatic Metastasis

Metastasis frequency was lowest in the ACN group (46%) compared to the other groups, which ranged from 60% to 69% (Supplementary Figure S3A and Supplementary Table S5). Although high variability within each group prevented statistical significance when comparing ACN to control, there was a trend toward a reduced number of breast cancer tumor nodules (Supplementary Figure S3B) and a smaller metastatic area (%) (Supplementary Figure S3C) in the ACN group. Consistently, metastatic grade distributions differed significantly among groups, with grade 4 metastasis absent in the control group (Supplementary Figure S3D).

#### 2.4.3. Cardiac Metastasis

BC metastasis frequency and incidence data (Supplementary Table S6) are shown in Supplementary Figure S4A. Significant differences were observed among groups, with ACN providing protection against cardiac metastasis—only two out of thirteen animals in this group showed positive heart metastasis. Among tissues with detected metastasis, the DOX-ACN group had a higher number of BC tumor nodules (Supplementary Figure S4B) yet exhibited a lower metastatic area percentage compared to DOX alone (Supplementary Figure S4C). Metastatic grade distribution also varied significantly across all groups (Supplementary Figure S4D).

#### 2.4.4. Renal Metastasis

BC metastasis in the kidneys is relatively uncommon but can impair kidney function. Animals were closely monitored for symptoms indicative of kidney metastasis, such as hematuria, fatigue, or weight loss, which could necessitate early termination. The ACN and DOX groups had the fewest animals with positive renal metastasis—only two out of thirteen animals each (Supplementary Figure S5A and Supplementary Table S7). While the total number of BC tumor nodules was similarly low in the ACN, DOX, and DOX-ACN groups compared to the control (Supplementary Figure S5B), the metastatic area (%) was higher in the DOX-ACN group relative to the others (Supplementary Figure S5C).

#### 2.4.5. Splenic Metastasis

Data on metastatic frequency and incidence in the spleen are summarized in Supplementary Table S8. In the control group, only one out of thirteen spleens analyzed showed BC metastasis, corresponding to a 7% frequency, while no metastasis was detected in the spleens of animals treated with ACN (Supplementary Figure S6A). These results were consistent with the total number of BC tumor nodules observed (Supplementary Figure S6B). The higher metastasis rates seen in the DOX (five out of thirteen animals) and DOX-ACN (four out of ten animals) groups may be linked to drug toxicity, unlike the ACN group, which showed no metastasis.

#### 2.4.6. CD3+ in Liver and Kidney Tissues

CD3+ immunohistochemistry (IHC) analysis in metastatic liver regions revealed no significant differences in CD3+ cell density or percentage of CD3+ area among groups (Supplementary Figure S7A,B), although values in the ACN group tended to be lower than in other groups. This trend was not observed when analyzing the total liver tissue area (Supplementary Figure S7C,D). Data from metastatic kidney regions were not analyzed due to limited sample size (only two animals with positive metastasis in both DOX and ACN groups). However, CD3+ cell density in total kidney tissue showed a significant difference between the DOX-ACN and control groups (Supplementary Figure S8A), while no differences were found in the percentage of CD3+ area (Supplementary Figure S8B).

## 3. Discussion

The BW results indicate that DOX treatment, similar to other chemotherapeutic agents, can induce BW loss or cachexia, characterized by severe loss of body weight, muscle, and fat. This condition may be accompanied by reversible side effects such as nausea, vomiting, diarrhea, stomatitis, mucositis, alopecia, and gastrointestinal disturbances, as well as potential long-term effects like cardiotoxicity [12]. However, the use of chemotherapeutic drugs requires weighing their potential life-saving and cancer-controlling benefits against the risks of adverse side effects. In this context, ACN and other polyphenols may help reduce or prevent these side effects by protecting healthy cells from chemotherapy-induced damage, partly through their immunomodulatory properties [13]. This could account for the absence of a statistically significant difference in BW between the control and DOX-ACN groups (*p* = 0.0692) (Figure 1A, lower panel).

With respect to tumor growth rates, the significant chemopreventive effect of ACN observed in this study contrasts with the pilot study [8], where ACN administration began only after tumor development had occurred and, as a result, failed to influence tumor growth compared to untreated controls [8]. Consequently, the results from this study strongly indicate that ACN may effectively delay BC tumor growth when intake is initiated before the onset of disease, such as by incorporating DSC into a regular diet.

The activity of anthocyanins against TNBC, as well as some of the underlying mechanisms involved in drug resistance, proliferation, and angiogenesis were reviewed by Rabelo et al., 2023 [14]. The STAT3 transcription factor plays a critical role in promoting BC metastasis by targeting genes that play a role in BC cell proliferation; survival and apoptosis inhibition (Bcl2, Bcl-xL, and survivin), angiogenesis (VEGF), invasion and metastasis (MMPs), EMT (Twist and Snail), inflammatory microenvironment that promote tumor growth and survival (IL-6) [15,16], and cellular response to hypoxia or low oxygen levels (HIF) [17]. The Snail family of transcription factors include Snail1 and Slug2, whose role in EMT is critical as it allows cancer cells to become more motile, invasive, and resistant to apoptosis [17].

HIF genes contribute to BC progression by regulating processes such as angiogenesis, metastasis, metabolic adaptation, therapy resistance, and influencing prognosis [18].

Overall, these findings are important because the interaction between STAT3 and HIF plays a key role in cancer and inflammation, with both pathways frequently upregulated. Suppressing their activity is recognized as a promising approach to inhibit metastasis and improve outcomes for BC patients.

The dysregulation of the mTOR signaling pathway is central to BC metastasis, influencing cell growth, proliferation, metastatic processes, angiogenesis, and therapy resistance, and is frequently associated with poor prognosis [19]. Numerous studies have highlighted the potential of natural products to target the PI3K-Akt-mTOR signaling pathway for cancer prevention and therapy [20].

These findings align with a previous pilot study showing the suppression of the mTOR pathway in 4T1 BC cells treated with ACN [8]. In the current study, mTOR mRNA levels paralleled those of STAT3 across experimental groups, reinforcing the close interplay between STAT3 and mTOR. This relationship is supported by mTOR’s ability to enhance STAT3 activity both by increasing the synthesis of proteins that activate STAT3 and by directly phosphorylating and activating Akt, which subsequently activates STAT3. Additionally, feedback loops exist wherein STAT3 upregulates components that further amplify mTOR signaling, thereby promoting tumor progression [19].

SIRTs are a family of NAD^+^-dependent deacetylases that can function as either tumor suppressors or promoters, depending on the cellular context. As a tumor promoter, SIRT1 is frequently overexpressed in BC and influences multiple pathways, including the inhibition of apoptosis, enhanced cell proliferation [21], angiogenesis [11], energy metabolism, reactive oxygen species regulation, and the maintenance of cancer stem cell characteristics—contributing to tumor initiation and metastatic progression [22]. As a result, dietary polyphenols are being investigated as potential SIRT inhibitors to improve the efficacy of cancer therapies, particularly in combating BC metastasis and multidrug resistance [22].

These findings are significant because, within the tumor microenvironment, TGFβ1 functions as an immunosuppressive cytokine that promotes immune evasion and supports tumor growth, angiogenesis, and metastasis [23].

IKKβ is a key regulator of nuclear factor kappa-light-chain-enhancer of activated B cells (NF-κB) activation in BC. Importantly, NF-κB activation is implicated in resistance to chemotherapy, radiation, and targeted therapies. Therefore, inhibiting NF-κB signaling, either through IKKβ or other upstream regulators, is an area of active research for overcoming drug resistance using phytomedicines [24].

Additionally, TGFβ expression has been identified as a downstream event of Cd44-dependent signaling, which is critical for tumor cell survival and the formation of metastatic colonies in the lung parenchyma of syngeneic mice [25]. The Cd44 gene encodes a cell surface glycoprotein that plays a crucial role in BC metastasis through its involvement in cell adhesion, EMT, cancer stem cell maintenance, survival, invasion, and immune evasion and is associated with aggressive tumor behavior and poor clinical outcomes. Cd44 also promotes a variety of functions independently or in cooperation with other cell-surface receptors through the activation of varied signaling pathways including the PI3K/AKT pathways to regulate cell adhesion, migration, survival, invasion, and EMT. It also regulates key pathways such as NF-κB, and CREB/TGFβ to drive BC progression [26] and interacts with EMT transcription factors such as Snail, enhancing their expression and activity [27]. Consequently, Cd44 is considered a promising target for the treatment of BC metastasis [28].

Lastly, Rgcc32 expression has been reported to be significantly associated with lung metastasis in TNBC patients [29]. This is supported by previous findings showing that targeting Rgcc32 in combination with chemotherapeutic agents effectively suppresses pulmonary metastasis in a TNBC mouse model [29]. In the current study, Rgcc32 mRNA expression followed a similar pattern to the transcription factor HIF (Figure 2C). Mechanistic studies have identified Rgcc32 as a hypoxia-inducible gene, suggesting its important homeostatic role in angiogenesis. Moreover, Rgcc32 is known to be upregulated by HIF-1α, promoting adaptive responses that facilitate tumor growth and survival under hypoxic conditions [30].

CREB has been linked to proteins involved in the acquisition of a metastatic phenotype in BC cells and is also associated with resistance to chemotherapy and other therapeutic interventions [13]. Its activation is regulated upstream by the PI3K/Akt signaling pathway, which is known to contribute to anti-apoptotic effects and drug resistance [31]. Consequently, PI3K and Akt-1 may represent additional molecular targets of ACN when used in combination with DOX treatment. This is particularly relevant, as dysregulation of the PI3K/Akt pathway has been implicated in resistance to DOX [31].

A study found that TNBC patients with positive Vim expression responded more favorably to chemotherapy compared to Vim-negative patients. Additionally, Vim has been identified as a potential therapeutic target capable of inhibiting BC progression and reducing resistance to hormone therapy-related drugs [32]. Thus, the observed upregulation of Vim in the ACN and DOX-ACN groups, compared to the DOX group, may offer benefits during advanced stages of BC metastasis [32]. These findings support further investigation into the potential of ACN as a complementary agent to DOX chemotherapy.

The findings related to Tjp are somewhat conflicting. While reduced Tjp expression can weaken cell–cell adhesion and promote a more migratory and invasive cancer cell phenotype—facilitating metastasis [33]—therapies targeting EMT may help maintain or restore Tjp function, thereby enhancing cell adhesion and restricting tumor spread. Interestingly, the downregulation of Tjp1 and its encoded protein, zonulin-1, is often accompanied by the decreased expression of other epithelial markers such as Cdh1. It is important to note that, in this study, metastasis to distant organs had already occurred by the time tumors were excised. Thus, evaluating Tjp gene expression at earlier stages of BC progression may provide more insight into its therapeutic relevance.

These results highlight the chemopreventive potential of ACN through its modulation of key genes involved in BC suppression. The findings also suggest that ACN may enhance the effectiveness of DOX treatment by targeting critical pathways, particularly those related to HIF, Cd44, Rgcc32, CREB, and PI3K/Akt-1. This is especially relevant given their association with more aggressive BC subtypes.

Overall, results from tumor gene expression analyses are supported by a previous study, in which cyanidin 3-O-glucoside (C-3G), also present in ACN extracts [7], significantly reduced the expression of metastasis-related genes when administered alone or in combination with a chemotherapeutic agent in a BALB/c nude mouse model bearing H661 large-cell lung carcinoma xenografts [34].

In terms of histopathological findings, the mitotic index grade serves as an important indicator of cancer progression by reflecting the rate of cell division within the tumor. The lack of statistically significant differences among experimental groups was expected, given that tumor excision occurred after cancer cells had already disseminated to distant organs and metastasis was established.

This study underscores the importance of investigating the effects of ACN on BC metastasis, the leading cause of mortality in BC patients. Flavonoids from plant-based diets have been shown to influence key processes that regulate metastasis, including angiogenesis, invasion, and cancer stemness, particularly in TNBC [35].

The lungs are a common site for TNBC metastasis, leading to high mortality as newly formed tumors impair lung function. Nevertheless, the exact molecular mechanisms driving lung-specific metastasis in TNBC remain poorly understood. The findings from this study support ACN’s protective effect against BC lung metastasis, consistent with previous results showing that ACN reduced lung mRNA levels of Cenpf [8], a gene associated with lung metastatic lesions and poor prognosis [14]. Additionally, the downregulation of Rgcc32 mRNA in tumors from the ACN group (Figure 2I) may also contribute to this protective effect. Given Rgcc32’s reported role in lung metastasis [29], its modulation by ACN may represent a key mechanism underlying ACN’s antimetastatic activity and warrants further investigation.

BC can metastasize to the liver at any stage of the disease. Naturally occurring compounds with antioxidant and anti-inflammatory properties show promise as adjuvant agents for protecting against hepatotoxicity in cancer patients and potentially lowering the risk of liver metastasis [36].

Notably, the DOX and DOX-ACN groups exhibited a greater number of BC tumor nodules and a higher metastatic area (%) in the liver compared to the ACN group. This may be attributed to DOX-induced liver inflammation, oxidative stress, and direct hepatotoxicity. Supporting this, a study involving BC patients undergoing chemotherapy with DOX, cyclophosphamide, and paclitaxel reported that advanced cancer stages were associated with significantly elevated oxidative stress markers and increased levels of malondialdehyde, a marker of lipid peroxidation. These findings suggest that chemotherapy may intensify oxidative stress, making the liver more vulnerable to damage and potentially promoting metastasis [37].

BC metastasis to the heart accounts for approximately 15.5% of all cardiac metastasis in humans [38]. Interestingly, the DOX and DOX-ACN groups showed similar frequency and incidence of cardiac metastasis compared to the control group, potentially reflecting the complex interplay between mitochondrial dysfunction and DOX-induced cardiotoxicity. However, the DOX-ACN group exhibited a lower metastatic area (%) than the DOX group (Supplementary Figure S4C). In this context, dietary antioxidants may support mitochondrial-targeted strategies to enhance the cardiac safety of DOX. This is reinforced by a study showing that the flavonoid luteolin prevented DOX-induced cardiotoxicity while simultaneously boosting its anticancer efficacy against TNBC by suppressing tumor growth and metastasis in a mouse model [39].

The kidneys are essential for filtering waste and eliminating drugs from the body. According to Gomez-Garduño et al., 2022 [40], anthocyanins may enhance the plasma concentration of DOX by modulating phase I drug-metabolizing enzymes and drug transporters. In 4T1 cells, ACN was also shown to regulate phase I enzymes, enhancing the antiproliferative efficacy of the DOX-ACN combination [41]. However, this modulation may also increase DOX-induced oxidative stress in the kidneys, potentially compromising renal function and contributing to metastasis, which could explain the trend toward a higher metastatic area in the DOX-ACN group (Supplementary Figure S5C). These findings raise concerns about the unregulated use of concentrated phytochemicals, such as those in dietary supplements, without appropriate oversight.

The spread of BC to spleen is relatively uncommon. However, certain chemotherapeutic agents and other aggressive treatments may exert toxic effects on the spleen, potentially leading to necrosis. Moreover, rapid tumor growth within the spleen can impair blood flow, causing substantial tissue damage. While preliminary findings suggest a potential protective effect of ACN against splenic metastasis, further research is needed to confirm this effect.

CD3+ cells are markers of T lymphocytes, which play a central role in adaptive immunity. Their presence in tissues indicates an immune response aimed at recognizing and eliminating abnormal or malignant cells. In the liver, CD3+ cells can have dual roles. During the early stages of disease, they contribute to tumor surveillance and elimination, and have been associated with improved outcomes in early-stage TNBC [3]. However, in advanced disease or under immunosuppressive conditions—such as those characterized by elevated TGFβ—T cells may either lose functionality or paradoxically support tumor progression by facilitating cancer cell seeding, colonization, and growth [42]. In the kidney, CD3+ cells may accumulate because of metastatic infiltration, chemotherapy-induced inflammation, or tissue toxicity.

In this study, the advanced metastatic stage in liver and kidney tissues made it difficult to draw definitive conclusions regarding CD3+ cell involvement. Nonetheless, evaluating CD3+ cell infiltration remains valuable for understanding how treatments like DOX and ACN influence the immune microenvironment. For instance, the increased CD3+ cell density in kidney tissue observed in the DOX-ACN group compared to control may reflect active immune surveillance or treatment-related inflammatory responses (Supplementary Figure S8A).

## 4. Materials and Methods

### 4.1. Chemicals and Reagents

India ink (nigrosin stain solution, 10% in water) was purchased from Sigma-Aldrich (St Louis, MO, USA); ammonium hydroxide 28–30 wt%, solvents used to extract the ACN extract, and 10% neutral buffered formalin (Azer brand) were purchased from Fisher Scientific (Houston, TX, USA). Isoflurane anesthesia (Attane brand) was purchased from the Comparative Medicine Program (CMP)’s laboratory at Texas A & M. Primers for interleukin (IL)-6, ribosomal protein L19 (RPL19), signal transducer and activator of transcription 3 (STAT3), CREB, PI3K, mTOR, I-kappa-B kinase beta (IKKβ), Nuclear Factor kappa-light-chain-enhancer of activated B cells (NF-κB), Sirtuin 1 (SIRT1), activated protein kinase alpha 2 (AMPKa2), matrix metallopeptidase (MMP)13, MMP7, B-cell lymphoma 2 (Bcl2), and β-actin were purchased from Integrated DNA Technologies (Coralville, IA, USA). Primers for Akt-1, E-cadherin (Cdh1), cluster of differentiation 44 (Cd44), hypoxia-inducible factor (HIF), transforming growth factor beta (TGFβ1), vimentin (Vim), tight junction protein 1 (Tjp1), response gene to complement (Rgcc)32, protein kinase AMP-activated catalytic subunit alpha 2 (Prkaa2), twist family BHLH transcription factor (Twist1), vascular endothelial growth factor A (VEGFA), IL-1β, Snail1, vimentin (Vim), and Cenpf, were purchased from Sigma-Aldrich.

### 4.2. Preparation of DSC Extract Rich in Anthocyanins (ACN)

The DSC concentrated juice utilized in this study was kindly supplied by FruitSmart, Inc (Grandview, WA, USA). The ACN prepared as reported [8] were dried in a rotavapor (Büchi, Switzerland) followed by vacuum centrifugation (Savant SpeedVac, Thermo Fisher Scientific, Asheville, NC, USA) at 48 °C, and dissolved with saline solution (SS) (0.9% sodium chloride sterile solution, BDF, Franklin Lakes, NJ, USA) to a known concentration of anthocyanins quantified as cyanidin-3-O-rutinoside equivalent (C3R) [43] for use in animal feeding.

### 4.3. Mouse Syngeneic Study

**Figure 8:**
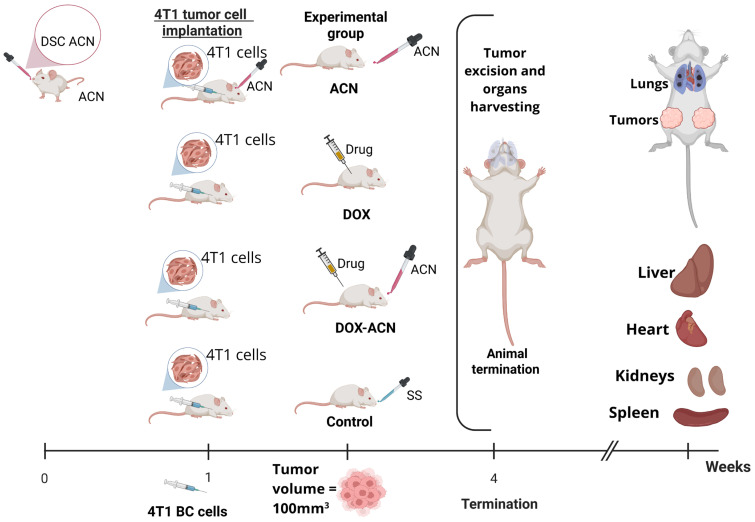
Experimental design. Created in Biorender.

BALB/C female mice purchased from Envigo (Houston, TX, USA) were housed at the CMP at Texas A&M University. The experimental design is illustrated in Figure 8. Briefly, upon arrival, mice were allowed to adjust to their environment for one week. All mice were maintained on a 12 h light–dark cycle with *ad libitum* access to food and filtered water. Mice were monitored daily for signs of pain or distress according to the guidelines of the Animal Research Advisory Committee published by the National Institutes of Health.

The 4T1 murine BC cells purchased from the American Type Culture Collection (ATCC, Manassas, VA, USA), were orthotopically injected into the 4th right and left mammary gland fat pads (0.5 × 10^6^ cells/pad) suspended in 50 μL of 30% Growth Factor Reduced Matrigel (BD Bioscience, San Jose, CA, USA) in sterile phosphate buffer solution (PBS) at pH 7.2 under isoflurane anesthesia. The animals’ responses during surgery were closely monitored to ensure they were at sufficient depth of anesthesia. Aseptic technique and conditions were ensured during tumor cell implantation. In brief, a bilateral skin incision was made between the 4th nipple and the midline to make a pocket and visualize the mammary fat pad. The skin incision was closed using Vetbond tissue adhesive (3M brand). Meloxicam (NSAID) at 1–2 mg/kg SQ injection was provided prior to surgery and once daily following surgery for at least 2 days to alleviate any signs of pain.

Mice in the ACN group were fed 150 mg C3R/kg body weight (BW) by gavage every other day starting 7 days before tumor cell implantation. Animals in the DOX group were injected with DOX (2 mg/kg BW) by intraperitoneal (i.p.) injection after tumor volume reached 100 mm^3^, and then every 4 days. Animals in DOX-ACN group started the ACN treatment (150 mg C3R/kg BW by gavage) every other day when the skin incision healed (2–4 days after tumor cell implantation), the DOX treatment after tumor volume reached 100 mm^3^, and then every 4 days. Mice in the control group were fed SS by gavage every other day. A total of *n* = 13 mice were assigned to each experimental group.

The tumor volumes were measured every 2 days using calipers. The shortest (a) and longest (b) perpendicular diameters of tumors were measured, wherein tumor volume was calculated as $\frac{a^{2}\times b}{2}$. Tumor growth rate was calculated as the average volume of the 2 tumors developed per mouse versus time. Day 1 was set for each mouse when any of the 2 implanted tumors reached 100 mm^3^. The body weight (BW) was measured every 4 days.

Animals were terminated between days 15 and 25 (15 to 25 days after the tumor reached 100mm^3^) when primary tumors grew to the limit volume (2000 mm^3^) or animals showed signs associated with pain or distress (e.g., impaired mobility, weight loss, respiratory distress, abnormal posture, hypoactivity, lethargy), as specified in the IACUC-approved protocol. Animals were euthanized by CO_2_ asphyxiation, followed by cervical dislocation. After euthanasia, tumors were excised, weighed, visually assessed for necrosis, sectioned to be preserved in a 10% neutral formalin buffer for histology, or in RNA*later* solution (Ambion, Thermo Fisher Scientific Inc. Waltham, MA, USA) to be stored at −80 °C for further gene expression analyses. Harvested organs (liver, heart, kidney, and spleen) were fixed in 10% buffered formalin for histological analysis. Experiments were approved by the Institutional Animal Care and Use Committee at Texas A & M, College Station, TX (IACUC 2022–0103, 18 August 2022).

### 4.4. Tumor Tissue Gene Expression Analyses

To increase the number of samples per experimental group while reducing variability associated with time, only the tumor tissues collected between days 17 and 23 (day 1 set when at least one tumor reached 100 mm^3^) were used in gene expression analyses.

Direct-zol™ RNA MiniPrep Plus (Zymo Research, Irvine, CA, USA) was used to extract mRNA from tumor tissues preserved in RNA*later* solution according to the manufacturer’s protocol. The reverse transcription supermix iScript^TM^ (Bio-Rad, Hercules, CA, USA) was used to synthesize cDNA from mRNA followed by amplification using SsoAdvanced™ Universal SYBR^®^ Green Supermix (Bio-Rad, Hercules, CA, USA) using specific pairs of primers to perform real-time polymerase chain reaction (RT-PCR). Relative mRNA levels were calculated by comparative C_T_ method as detailed [44]. Mouse ribosomal protein L19 (RPL19) and β-actin were tested as housekeeping genes, but only RPL19 was selected due to the minimal inter-sample variability. Primer sequences are presented in Supplementary Table S9.

### 4.5. Lung Metastasis

Among the symptoms of lung metastasis are shortness of breath, fatigue, weight loss, and difficulty breathing. Mice were closely monitored for these symptoms, which were used as a criterion for early euthanasia. India ink inflation was performed postmortem to visualize lung metastasis, following the protocol described by Paschall and Liu, 2016 [45]. Briefly, after euthanasia, mice were securely placed on their backs, and a thoracotomy was performed by cutting through the rib cage to expose the lungs. Approximately 1 mL of India ink solution (10% India ink and 0.1% Ammonium Hydroxide) was injected through the trachea using a ½ CC 27 G tuberculin syringe. Metastatic tumor nodules do not absorb the India ink as lungs do; therefore, they remained white. Lung lobes were removed with forceps, rinsed, and stored in Fekete’s solution (50% ethanol, 6% formaldehyde, and 3% glacial acetic acid) at 4 °C. Lungs were photographed using a Canon EOS 80D camera with EF-S 60 mm Macro lens. Pictures were analyzed using ImageJ 1.54p to quantify total tissue area with metastasis.

### 4.6. Histopathological Analysis of Tumors and Organs

Histopathological analyses were performed only in animals terminated between days 17 and 23 to analyze the highest number of experimental units and to decrease the influence of time on the metastasis progress.

#### Tumors, Liver, Heart, Kidneys, and Spleen

Tissue sections preserved in 10% neutral buffered formalin were routinely processed and embedded in paraffin, sectioned at 5 µm, with at least four sections per tumor or organ, and stained with hematoxylin and eosin (H&E). All formalin-preserved tissues were analyzed in a blind manner by a board-certified veterinary anatomic pathologist (LWS).

a. Tumor mitosis and necrosis. The mitotic rate of each primary tumor was calculated by counting the total number of mitotic figures in ten 400× fields.

The necrotic areas in tumor tissues were determined using ImageJ to calculate the percentage of tissue area affected by necrosis relative to the total area of the H&E-stained tumor tissue section.

Images were captured at 2× magnification using an Olympus BX53F2 microscope and LC35 color camera equipped with cellSens software v 4.4. Images were analyzed using QuPath Bioimage analysis v. 0.4.3.

b. Metastasis. Organs were visually assessed for presence (+) or absence (−) of metastasis. Additionally, the total number of metastatic tumor nodules visualized on the external organ surface and in H&E-stained tissue sections were visually determined. The metastatic area relative to the total area of the H&E-stained organ tissue section was quantified using ImageJ.

c. CD3+ immunohistochemistry and digital image analysis. The paraffin-embedded liver and kidney tissue sections of 5 µm were processed for immunohistochemical (IHC) staining using a protocol optimized by the Veterinary Medicine Biomediacal Sciences (VMBS) core histology lab at Texas A & M University (TAMU). The antibody against CD3+ (dilution 1:200) (Dako Polyclonal Rabbit Anti-mouse CD3+, A0452), was purchased from Agilent Technologies (Santa Clara, CA, USA); the conjugated goat anti-rabbit polymer-horseradish peroxidase secondary antibody was purchased from Biocare Medical (Pacheco, CA, USA). Detection was performed with the Chromogen, ImmPact DAB (SK_4105) kit purchased from Vector laboratories (Newark, CA, USA), followed by counter staining with hematoxylin (CAT Hematoxylin, Biocare Medical).

Digital images of the stained tissue sections were obtained at 20× magnification using the Aperio CS2 5-slide scanner (Leica Biosystems Imaging, Inc. Deer Park, IL, USA). The brightfield digital images were analyzed for the presence of CD3+ cells in sections of harvested organs using Visiopharm^®^ software 2025.02.1 (Broomfield, CO, USA). In brief, metastatic regions and total tissue sections were annotated, and CD3+ area (µm^2^) and cell count within metastatic and total tissue regions were quantified using the Visiopharm^®^ software. The percentage of CD3+ cells and density (cells/mm^2^) were then calculated for both metastatic and total tissue areas.

### 4.7. Statistical Analyses

Data from tumor growth rate and BW was analyzed by two-way ANOVA full model (treatment effect, time effect, and interaction effect), followed by Tukey’s multiple comparison test after passing the D’Agostino & Pearson normality test.

#### 4.7.1. Tumor Tissues

Data from gene expression in BC tumor tissues was subjected to outlier detection by the ROUT method (Q = 10%). The one-way ANOVA of cleaned data was followed by Tukey’s multiple comparisons test if normally distributed; otherwise the Kruskal–Wallis test followed by Dunn’s multiple comparisons test with p-values ≤ 0.05 considered statistically significant was used. Heatmap cluster visualization was performed with MetaboAnalyst 6.0.

Data from tumor gene expression was subjected to Spearman correlation analysis with *p* value (two tail) considered significant (*) for α ≤ 0.05, highly significant (**) for α ≤ 0.01, and very highly significant (***) for α ≤ 0.001 or (****) for α ≤ 0.0001, respectively. Heatmap of Spearman correlations were performed with R 4.5.

Data from tumor necrosis area and mitotic index was analyzed by one-way ANOVA, followed by uncorrected Fisher’s LSD test. Grades of necrosis grade and mitotic index were determined by transforming data into fractions of total from all experimental groups followed by descriptive statistical analysis to determine minimum, maximum, and interquartile values. Tumor necrosis and mitotic index grades were assigned as 1 (from minimum value to <25% percentile), 2 (from 25% percentile to < median), 3 (from median to < 75% percentile), and 4 (from 75% percentile to maximum value). Frequency distributions of tumor necrosis and mitotic index grades were determined for each experimental group and statistical differences between groups were determined by the chi-square test to determine statistical differences between groups.

#### 4.7.2. Organs

Data from presence (+) or absence (−) of BC metastatic tumor nodules was used to determine metastatic frequency (%), and incidence (fraction of total number of animals analyzed per experimental group (*N*). Statistical differences between experimental groups in frequency distributions (% of (+) metastasis and (−) metastasis) were determined by the chi-square test.

Metastatic grades were determined by transforming data into fractions of total from all experimental groups followed by descriptive statistical analysis to determine minimum, maximum, and interquartile values. Metastatic grade was assigned as 1 (from minimum value to <25% percentile), 2 (from 25% percentile to <median), 3 (from median to <75% percentile), and 4 (from 75% percentile to maximum value). The distribution of metastatic grades determined as frequency distributions per experimental group were analyzed by the chi-square test to determine statistical differences between groups.

Data from total number of BC tumor nodules and tissue area with metastasis were subjected to outlier detection by the ROUT method (Q = 10%). The one-way ANOVA of cleaned data followed by Fisher’s LSD multiple comparisons test were performed if normality and equal variances assumptions were met; otherwise Kruskal–Wallis test followed by uncorrected Dunn’s multiple comparisons test with *p*-values ≤ 0.05 (*), ≤ 0.01 (**), ≤ 0.001 (***), ≤ 0.0001 (****) was used. Statistical analyses were performed using GraphPad Prism software version 9.0.0 for windows (Boston, MA, USA).

## 5. Conclusions

This study demonstrated that ACN intake, initiated one week prior to tumor cell implantation, effectively delayed TNBC tumor growth without signs of toxicity, as evidenced by BW gain throughout the intervention. While DOX treatment impaired BW gain, it significantly delayed tumor growth only at later stages (days 10–12). In contrast, ACN administered as a phyto-adjuvant to DOX significantly inhibited tumor growth from days 7 to 12 and supported BW gain.

Gene expression analysis in tumor tissues revealed that ACN, whether used alone or in combination with DOX, significantly downregulated several key genes associated with BC metastasis, such as HIF, Cd44, Rgcc32, CREB, PI3K/Akt-1, which were not effectively suppressed by DOX alone.

Moreover, strong correlations between mRNA levels observed in the control and DOX groups were absent in the ACN and DOX-ACN groups, highlighting Cd44, mTOR, Rgcc32, SIRT1, Snail1, and TGFβ1 as potential ACN targets.

In addition, significant gene–gene correlations involving CREB, PI3K, Akt-1, and Vim seen in the DOX group were disrupted in the DOX-ACN group, suggesting that ACN may interfere with critical molecular interactions, thereby enhancing the efficacy of DOX.

**Figure 9:**
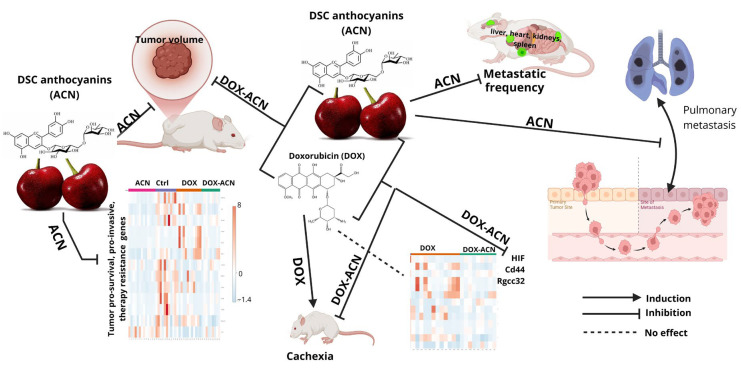
Summary of mechanisms of action of ACN as chemopreventive intervention and a phyto-adjuvant to DOX treatment.

As a chemopreventive agent, ACN significantly reduced lung metastasis compared to both control and DOX groups. When combined with DOX, ACN further decreased lung metastatic burden to levels comparable to ACN alone. ACN intake also reduced metastatic frequency in the liver, heart, kidneys, and spleen. However, the total number of tumor nodules and metastatic area (%) in metastasis-positive organs did not always reach statistical significance, likely due to high inter-animal variability. Figure 9 summarizes the main findings of this study, illustrating the effects of ACN as a chemopreventive agent and a phyto-adjuvant in the DOX-ACN treatment.

A key limitation of the study is the absence of data on metastatic markers in tumors during earlier stages of tumor progression before metastasis was established. Future research is needed to further explore the potential of ACN as a phyto-adjuvant to chemotherapy or targeted therapy, particularly in overcoming resistance and improving clinical outcomes.

## Data Availability

Request to access the datasets should be directed to gnoratto@tamu.edu.

## Supplementary Materials

The following supporting information can be downloaded at https://www.mdpi.com/article/10.3390/ijms26157225/s1.

## References

[1] World Health Organization, Breast Cancer. Key Facts 2025. https://www.who.int/news-room/fact-sheets/detail/breast-cancer.

[2] Rapoport B.L., Nayler S., Mlecnik B., Smit T., Heyman L., Bouquet I., Martel M., Galon J., Benn C.A., Anderson R. (2022). Tumor-Infiltrating Lymphocytes (TILs) in Early Breast Cancer Patients: High CD3, CD8, and Immunoscore Are Associated with a Pathological Complete Response. Cancers.

[3] Denkert C., von Minckwitz G., Darb-Esfahani S., Lederer B., Heppner B.I., Weber K.E., Budczies J., Huober J., Klauschen F., Furlanetto J. (2018). Tumour-infiltrating lymphocytes and prognosis in different subtypes of breast cancer: A pooled analysis of 3771 patients treated with neoadjuvant therapy. Lancet Oncol..

[4] Wu T.N., Chen H.M., Shyur L.F. (2021). Current Advancements of Plant-Derived Agents for Triple-Negative Breast Cancer Therapy through Deregulating Cancer Cell Functions and Reprogramming Tumor Microenvironment. Int. J. Mol. Sci..

[5] Koh Y.-C., Ho C.-T., Pan M.-H. (2020). Recent advances in cancer chemoprevention with phytochemicals. J. Food Drug Anal..

[6] Malavia N., Kuche K., Ghadi R., Jain S. (2021). A bird’s eye view of the advanced approaches and strategies for overshadowing triple negative breast cancer. J. Control. Release.

[7] Lage N.N., Layosa M.A.A., Arbizu S., Chew B.P., Pedrosa M.L., Mertens-Talcott S., Talcott S., Noratto G.D. (2020). Dark sweet cherry (Prunus avium) phenolics enriched in anthocyanins exhibit enhanced activity against the most aggressive breast cancer subtypes without toxicity to normal breast cells. J. Funct. Foods.

[8] Silveira Rabelo A.C., Mertens-Talcott S.U., Chew B.P., Noratto G. (2022). Dark Sweet Cherry (Prunus avium) Anthocyanins Suppressed ERK1/2-Akt/mTOR Cell Signaling and Oxidative Stress: Implications for TNBC Growth and Invasion. Molecules.

[9] Zhang H.P., Jiang R.Y., Zhu J.Y., Sun K.N., Huang Y., Zhou H.H., Zheng Y.B., Wang X.J. (2024). PI3K/AKT/mTOR signaling pathway: An important driver and therapeutic target in triple-negative breast cancer. Breast Cancer.

[10] Sun J., Huang J., Lan J., Zhou K., Gao Y., Song Z., Deng Y., Liu L., Dong Y., Liu X. (2019). Overexpression of CENPF correlates with poor prognosis and tumor bone metastasis in breast cancer. Cancer Cell Int..

[11] Carafa V., Altucci L., Nebbioso A. (2019). Dual Tumor Suppressor and Tumor Promoter Action of Sirtuins in Determining Malignant Phenotype. Front. Pharmacol..

[12] Mattioli R., Ilari A., Colotti B., Mosca L., Fazi F., Colotti G. (2023). Doxorubicin and other anthracyclines in cancers: Activity, chemoresistance and its overcoming. Mol. Aspects Med..

[13] Zheng T., Huang J., Xiang X., Li S., Yu J., Qu K., Xu Z., Han P., Dong Z., Liu Y. (2021). Systematical analysis reveals a strong cancer relevance of CREB1-regulated genes. Cancer Cell Int..

[14] Rabelo A.C.S., Guerreiro C.A., Shinzato V.I., Ong T.P., Noratto G. (2023). Anthocyanins Reduce Cell Invasion and Migration through Akt/mTOR Downregulation and Apoptosis Activation in Triple-Negative Breast Cancer Cells: A Systematic Review and Meta-Analysis. Cancers.

[15] To S.Q., Dmello R.S., Richards A.K., Ernst M., Chand A.L. (2022). STAT3 Signaling in Breast Cancer: Multicellular Actions and Therapeutic Potential. Cancers.

[16] Ma J.H., Qin L., Li X. (2020). Role of STAT3 signaling pathway in breast cancer. Cell Commun. Signal.

[17] Wong G.L., Manore S.G., Doheny D.L., Lo H.W. (2022). STAT family of transcription factors in breast cancer: Pathogenesis and therapeutic opportunities and challenges. Semin. Cancer Biol..

[18] Mirzaei S., Ranjbar B., Tackallou S.H., Aref A.R. (2023). Hypoxia inducible factor-1alpha (HIF-1alpha) in breast cancer: The crosstalk with oncogenic and onco-suppressor factors in regulation of cancer hallmarks. Pathol. Res. Pract..

[19] Malayil R., Chhichholiya Y., Vasudeva K., Singh H.V., Singh T., Singh S., Munshi A. (2023). Oncogenic metabolic reprogramming in breast cancer: Focus on signaling pathways and mitochondrial genes. Med. Oncol..

[20] Tewari D., Patni P., Bishayee A., Sah A.N., Bishayee A. (2022). Natural products targeting the PI3K-Akt-mTOR signaling pathway in cancer: A novel therapeutic strategy. Semin. Cancer Biol..

[21] Onyiba C.I., Scarlett C.J., Weidenhofer J. (2022). The Mechanistic Roles of Sirtuins in Breast and Prostate Cancer. Cancers.

[22] Sinha S., Sharma S., Vora J., Shrivastava N. (2020). Emerging role of sirtuins in breast cancer metastasis and multidrug resistance: Implication for novel therapeutic strategies targeting sirtuins. Pharmacol. Res..

[23] Shukla N., Naik A., Moryani K., Soni M., Shah J., Dave H. (2022). TGF-beta at the crossroads of multiple prognosis in breast cancer, and beyond. Life Sci..

[24] Ansari J.A., Malik J.A., Ahmed S., Bhat F.A., Khanam A., Mir S.A., Abouzied A.S., Ahemad N., Anwar S. (2023). Targeting Breast Cancer Signaling via Phytomedicine and Nanomedicine. Pharmacology.

[25] Yu Q., Stamenkovic I. (2004). Transforming growth factor-beta facilitates breast carcinoma metastasis by promoting tumor cell survival. Clin. Exp. Metastasis.

[26] Ouhtit A., Rizeq B., Saleh H.A., Rahman M.M., Zayed H. (2018). Novel CD44-downstream signaling pathways mediating breast tumor invasion. Int. J. Biol. Sci..

[27] Mimeault M., Batra S.K. (2014). Molecular biomarkers of cancer stem/progenitor cells associated with progression, metastases, and treatment resistance of aggressive cancers. Cancer Epidemiol. Biomarkers Prev..

[28] Al-Othman N., Alhendi A., Ihbaisha M., Barahmeh M., Alqaraleh M., Al-Momany B.Z. (2020). Role of CD44 in breast cancer. Breast Dis..

[29] Cheng S., Wan X., Yang L., Qin Y., Chen S., Liu Y., Sun Y., Qiu Y., Huang L., Qin Q. (2023). RGCC-mediated PLK1 activity drives breast cancer lung metastasis by phosphorylating AMPKalpha2 to activate oxidative phosphorylation and fatty acid oxidation. J. Exp. Clin. Cancer Res..

[30] An X., Jin Y., Guo H., Foo S.Y., Cully B.L., Wu J., Zeng H., Rosenzweig A., Li J. (2009). Response gene to complement 32, a novel hypoxia-regulated angiogenic inhibitor. Circulation.

[31] McCubrey J.A., Steelman L.S., Abrams S.L., Lee J.T., Chang F., Bertrand F.E., Navolanic P.M., Terrian D.M., Franklin R.A., D’Assoro A.B. (2006). Roles of the RAF/MEK/ERK and PI3K/PTEN/AKT pathways in malignant transformation and drug resistance. Adv. Enzyme Regul..

[32] Chen Z., Fang Z., Ma J. (2021). Regulatory mechanisms and clinical significance of vimentin in breast cancer. Biomed. Pharmacother..

[33] Bazzoun D., Lelievre S., Talhouk R. (2013). Polarity proteins as regulators of cell junction complexes: Implications for breast cancer. Pharmacol. Ther..

[34] Wu C.F., Wu C.Y., Lin C.F., Liu Y.W., Lin T.C., Liao H.J., Chang G.R. (2022). The anticancer effects of cyanidin 3-O-glucoside combined with 5-fluorouracil on lung large-cell carcinoma in nude mice. Biomed. Pharmacother..

[35] Malla R.R., Deepak K.G.K., Merchant N., Dasari V.R. (2020). Breast Tumor Microenvironment: Emerging target of therapeutic phytochemicals. Phytomedicine.

[36] Mahmoudi F., Arasteh O., Elyasi S. (2023). Preventive and therapeutic use of herbal compounds against doxorubicin induced hepatotoxicity: A comprehensive review. Naunyn-Schmiedeb. Arch. Pharmacol..

[37] Sri Phani T.P., Mannangatti M., Nekkala R., Bellala V.M., Bellala R.S., Payala V. (2022). Oxidative stress in breast cancer after chemotherapy. Bioinformation.

[38] Kopcik K., Koscielecka K., Krzyzak K. (2023). Cardiac Metastatic Tumors: Current Knowledge. Am. J. Clin. Oncol..

[39] Shi Y., Li F., Shen M., Sun C., Hao W., Wu C., Xie Y., Zhang S., Gao H., Yang J. (2021). Luteolin Prevents Cardiac Dysfunction and Improves the Chemotherapeutic Efficacy of Doxorubicin in Breast Cancer. Front. Cardiovasc. Med..

[40] Gomez-Garduno J., Leon-Rodriguez R., Alemon-Medina R., Perez-Guille B.E., Soriano-Rosales R.E., Gonzalez-Ortiz A., Chavez-Pacheco J.L., Solorio-Lopez E., Fernandez-Perez P., Rivera-Espinosa L. (2022). Phytochemicals That Interfere With Drug Metabolism and Transport, Modifying Plasma Concentration in Humans and Animals. Dose Response.

[41] Nava-Ochoa A., Mertens-Talcott S.U., Talcott S.T., Noratto G.D. (2025). Dark Sweet Cherry (L.) Juice Phenolics Rich in Anthocyanins Exhibit Potential to Inhibit Drug Resistance Mechanisms in 4T1 Breast Cancer Cells via the Drug Metabolism Pathway. Curr. Issues Mol. Biol..

[42] Tabaries S., Ouellet V., Hsu B.E., Annis M.G., Rose A.A., Meunier L., Carmona E., Tam C.E., Mes-Masson A.M., Siegel P.M. (2015). Granulocytic immune infiltrates are essential for the efficient formation of breast cancer liver metastases. Breast Cancer Res..

[43] Giusti M.M., Wrolstad R.E. (2001). Characterization and Measurement of Anthocyanins by UV-Visible Spectroscopy. Curr. Protoc. Food Anal. Chem..

[44] Schmittgen T.D., Livak K.J. (2008). Analyzing real-time PCR data by the comparative C(T) method. Nat. Protoc..

[45] Paschall A.V., Liu K. (2016). An Orthotopic Mouse Model of Spontaneous Breast Cancer Metastasis. J. Vis. Exp..

