# Supplementary material for: Dark Sweet Cherry Anthocyanins Suppressed Triple-Negative Breast Cancer Pulmonary Metastasis and Downregulated Genes Associated with Metastasis and Therapy Resistance In Vivo
Source: Int J Mol Sci. 2025 Jul 25;26(15):7225. doi: 10.3390/ijms26157225 (PMC12346644; doi:10.3390/ijms26157225)
Supplement: Supplementary file 1 [file ijms-26-07225-s001.zip › ijms-3737601-supplementary.pdf]

International Journal of *Molecular Sciences*

MDPI

Supplementary Table S1. mRNA levels of genes associated with cell survival, therapy resistance, invasion, migration, EMT, stem cell properties, immunosuppression, and metastasis.

	Mechanisms	Gene	Fold of Control ± SEM		Comparisons	Mean Diff. or mean rank Diff.	Summary	*P* value	*N1*	*N2*
Transcription Factor	EMT, promotion of invasion and migration, Stemness associated with therapy resistance, angiogenesis, immunosuppression, interaction with signaling pathways involved in cancer progression	STAT3	Control	1.00 ± 0.295	Control vs. DOX	0.001675	***	0.0008	8	10
			ACN	0.12 ± 0.029	Control vs. DOX-ACN	0.001588	**	0.0020	8	9
			DOX	0.18 ± 0.064	Control vs. ACN	0.001806	***	0.0002	8	12
			DOX-ACN	0.23 ± 0.0691	DOX vs. DOX-ACN	−8.725e−005	ns	0.9957	10	9
					DOX vs. ACN	0.0001314	ns	0.9824	10	12
					DOX-ACN vs. ACN	0.0002186	ns	0.9319	9	12
Transcription Factor	EMT, invasion and migration, resistance to apoptosis and anoikis, stemness, angiogenesis, interaction with signaling pathways involved in cancer progression, prognostic factor	Snail1	Control	1.00 ± 0.354	Control vs. DOX	0.0001301	**	0.0051	8	11
			ACN	0.23 ± 0.0524	Control vs. DOX-ACN	0.0001076	*	0.0355	8	9
			DOX	0.23 ± 0.0456	Control vs. ACN	0.0001302	**	0.0036	8	13
			DOX-ACN	0.36 ± 0.0611	DOX vs. DOX-ACN	−2.249e−005	ns	0.9181	11	9
					DOX vs. ACN	1.547e−007	ns	>0.9999	11	13
					DOX-ACN vs. ACN	2.265e−005	ns	0.9082	9	13
Transcription Factor	Tumor growth, survival, EMT, resistance to therapy, angiogenesis	CREB[#]	Control	1.00 ± 0.288	Control vs. DOX	−6.646	ns	>0.9999	8	12
			ACN	0.24 ± 0.0395	Control vs. DOX-ACN	2.259	ns	>0.9999	8	7
			DOX	4.80 ± 1.58	Control vs. ACN	12.64	ns	0.0830	8	10
			DOX-ACN	0.70 ± 0.214	DOX vs. DOX-ACN	8.905	ns	0.5018	12	7
					DOX vs. ACN	19.28	***	0.0002	12	10
					DOX-ACN vs. ACN	10.38	ns	0.3100	7	10
Transcription Factor	Angiogenesis, EMT, metabolic reprogramming	HIF	Control	1.00 ± 0.176	Control vs. DOX	0.1750	ns	>0.9999	10	12
			ACN	0.24 ± 0.0354	Control vs. DOX-ACN	14.43	ns	0.0926	10	8
			DOX	1.17 ± 0.273	Control vs. ACN	18.42	**	0.0029	10	13
			DOX-ACN	0.34 ± 0.0778	DOX vs. DOX-ACN	14.25	ns	0.0774	12	8
					DOX vs. ACN	18.24	**	0.0017	12	13
					DOX-ACN vs. ACN	3.990	ns	>0.9999	8	13
	Cell growth, proliferation, and survival, regulation of EMT, regulation of cytoskeletal dynamics, cell adhesion molecules, proteolytic enzymes, angiogenesis, resistance to therapy	PI3K[#]	Control	1.00 ± 0.520	Control vs. DOX	-8.583	ns	0.5437	8	12
			ACN	0.35 ± 0.154	Control vs. DOX-ACN	-3.143	ns	>0.9999	8	7
			DOX	6.94 ± 2.42	Control vs. ACN	6.182	ns	>0.9999	8	11
			DOX-ACN	1.29 ± 0.589	DOX vs. DOX-ACN	5.440	ns	>0.9999	12	7
					DOX vs. ACN	14.77	**	0.0087	12	11
					DOX-ACN vs. ACN	9.325	ns	0.4960	7	11
		Akt-1[#]	Control	1.00 ± 0.163	Control vs. DOX	−0.6162	ns	>0.9999	9	11

 https://doi.org/10.3390/ijms26157225

Mechanisms	Gene	Fold of Control ± SEM		Comparisons	Mean Diff. or mean rank Diff.	Summary	*P* value	*N*1	*N*2
		ACN	0.407 ± 0.108	Control vs. DOX-ACN	3.049	ns	>0.9999	9	8
		DOX	1.52 ± 0.361	Control vs. ACN	12.97	ns	0.0681	9	11
		DOX-ACN	0.96 ± 0.266	DOX vs. DOX-ACN	3.665	ns	>0.9999	11	8
				DOX vs. ACN	13.59	*	0.0311	11	11
				DOX-ACN vs. ACN	9.926	ns	0.3658	8	11
Growth, proliferation, metabolism, and survival, regulation of pathways involved in EMT, angiogenesis, resistance to therapy, prognostic marker	mTOR	Control	1.00 ± 0.261	Control vs. DOX	0.001096	**	0.0041	9	9
		ACN	0.32 ± 0.0719	Control vs. DOX-ACN	0.0008507	*	0.0345	9	9
		DOX	0.27 ± 0.0788	Control vs. ACN	0.0009909	**	0.0049	9	13
		DOX-ACN	0.42 ± 0.0947	DOX vs. DOX-ACN	-0.0002452	ns	0.8437	9	9
				DOX vs. ACN	-0.0001049	ns	0.9807	9	13
				DOX-ACN vs. ACN	0.0001403	ns	0.9559	9	13
Metastasis, EMT, survival, invasion, and immune evasion, self-renewal capabilities	Cd44	Control	1.00 ± 0.241	Control vs. DOX	0.007192	ns	0.7309	10	10
		ACN	0.16 ± 0.0231	Control vs. DOX-ACN	0.02280	*	0.0145	10	9
		DOX	0.76 ± 0.231	Control vs. ACN	0.02513	**	0.0024	10	13
		DOX-ACN	0.23 ± 0.0611	DOX vs. DOX-ACN	0.01561	ns	0.1462	10	9
				DOX vs. ACN	0.01794	*	0.0440	10	13
				DOX-ACN vs. ACN	0.002331	ns	0.9856	9	13
EMT, migration, invasion, extra cellular matrix remodeling, immunosuppression, angiogenesis, and interaction with the tumor microenvironment	TGFβ1	Control	1.00 ± 0.338	Control vs. DOX	0.004154	*	0.0235	9	8
		ACN	0.14 ± 0.0283	Control vs. DOX-ACN	0.004389	*	0.0120	9	9
		DOX	0.25 ± 0.0823	Control vs. ACN	0.004745	**	0.0031	9	12
		DOX-ACN	0.21 ± 0.0585	DOX vs. DOX-ACN	0.0002348	ns	0.9982	8	9
				DOX vs. ACN	0.0005907	ns	0.9678	8	12
				DOX-ACN vs. ACN	0.0003559	ns	0.9918	9	12
Cell survival, inflammation and tumor microenvironment, EMT, therapeutic resistance	IKKβ	Control	1.00 ± 0.318	Control vs. DOX	17.07	*	0.0112	10	11
		ACN	0.12 ± 0.0261	Control vs. DOX-ACN	9.689	ns	0.5585	10	9
		DOX	0.12 ± 0.0418	Control vs. ACN	14.57	*	0.0348	10	13
		DOX-ACN	0.21 ± 0.0632	DOX vs. DOX-ACN	−7.384	ns	>0.9999	11	9
				DOX vs. ACN	−2.503	ns	>0.9999	11	13
				DOX-ACN vs. ACN	4.880	ns	>0.9999	9	13
EMT, modulation of metastatic pathways, angiogenesis, metabolic processes, prognostic marker	SIRT1	Control	1.00 ± 0.190	Control vs. DOX	0.0007594	**	0.0035	10	10
		ACN	0.21 ± 0.0362	Control vs. DOX-ACN	0.0008179	**	0.0021	10	9
		DOX	0.54 ± 0.236	Control vs. ACN	0.0009188	***	0.0002	10	12
		DOX-ACN	0.29 ± 0.0719	DOX vs. DOX-ACN	5.856e−005	ns	0.9922	10	9
				DOX vs. ACN	0.0001594	ns	0.8460	10	12
				DOX-ACN vs. ACN	0.0001008	ns	0.9581	9	12
	Rgcc32	Control	1.00 ± 0.201	Control vs. DOX	−0.0001321	ns	0.9990	10	10

**Mechanisms**	**Gene**	**Fold of Control ± SEM**		**Comparisons**	**Mean Diff. or mean rank Diff.**	**Summary**	***P* value**	***N1***	***N2***
Cell proliferation, EMT, migration and invasion, angiogenesis, interaction with PI3K/Akt and MAPK signaling pathway		ACN	0.53 ± 0.0593	Control vs. DOX-ACN	0.003446	**	0.0080	10	8
		DOX	1.02 ± 0.0659	Control vs. ACN	0.002768	*	0.0186	10	13
		DOX-ACN	0.42 ± 0.0579	DOX vs. DOX-ACN	0.003578	**	0.0056	10	8
				DOX vs. ACN	0.002900	*	0.0127	10	13
				DOX-ACN vs. ACN	−0.0006785	ns	0.8922	8	13
EMT, migration, invasion, prognostic factor, therapeutic resistance	Vim#	Control	1.00 ± 0.240	Control vs. DOX	2.096	ns	0.0767	10	10
		ACN	1.07 ± 0.187	Control vs. DOX-ACN	0.7505	ns	0.8202	10	9
		DOX	0.33 ± 0.112	Control vs. ACN	−0.2233	ns	0.9920	10	13
		DOX-ACN	0.76 ± 0.166	DOX vs. DOX-ACN	−1.345	ns	0.4134	10	9
				DOX vs. ACN	−2.319	*	0.0275	10	13
				DOX-ACN vs. ACN	−0.9739	ns	0.6330	9	13
Modulation of signaling pathways, EMT, prognostic marker.	Tjp	Control	1.00 ± 0.206	Control vs. DOX	0.004343	**	0.0023	10	11
		ACN	0.19 ± 0.0316	Control vs. DOX-ACN	0.004758	**	0.0020	10	8
		DOX	0.35 ± 0.101	Control vs. ACN	0.005444	****	<0.0001	10	13
		DOX-ACN	0.29 ± 0.0881	DOX vs. DOX-ACN	0.0004146	ns	0.9854	11	8
				DOX vs. ACN	0.001101	ns	0.7237	11	13
				DOX-ACN vs. ACN	0.0006864	ns	0.9331	8	13

Relative mRNA levels were calculated using the comparative CT method, with RPL19 serving as the housekeeping gene. Tumor tissues were collected from animals euthanized between timepoints 9 and 12. Mean Diff. or mean rank Diff. were calculated from fold change values ($2^{\wedge\Delta\Delta cq}$). Statistical analysis was conducted using one-way ANOVA followed by Tukey's multiple comparisons test for normally distributed data; otherwise, the Kruskal-Wallis test followed by Dunn's multiple comparisons test was used (#),(ns, $p > 0.05$), (*, $p \leq 0.05$), (**, $p \leq 0.01$), (***, $p \leq 0.001$), (****, $p \leq 0.0001$). *N1* and *N2* represent the total number of samples for each experimental group after outliers were removed by the ROUT method (Q = 10%).

Supplementary Table S2. mRNA levels of genes associated with cell survival, therapy resistance, invasion, migration, EMT, stem cell properties, immunosuppression, and metastasis.

Gene	Comparisons	Mean Diff. or Mean Rank Diff.	*P* value	*N1*	*N2*
Cdh1[#]	Control vs. DOX	12.15	0.1610	10	11
	Control vs. DOX-ACN	13.60	0.1104	10	9
	Control vs. ACN	12.06	0.1343	10	13
	DOX vs. DOX-ACN	1.455	>0.9999	11	9
	DOX vs. ACN	−0.08392	>0.9999	11	13
	DOX-ACN vs. ACN	−1.538	>0.9999	9	13
Cenpf	Control vs. DOX	0.0006358	0.8336	9	11
	Control vs. ACN-DOX	0.0005234	0.9107	9	9
	Control vs. ACN	0.001283	0.3068	9	13
	DOX vs. ACN-DOX	−0.0001124	0.9988	11	9
	DOX vs. ACN	0.0006467	0.7832	11	13
	ACN-DOX vs. ACN	0.0007591	0.7253	9	13
Prkaa[#]	Control vs. DOX	6.375	0.9649	8	8
	Control vs. ACN-DOX	12.33	0.1045	8	5
	Control vs. ACN	4.625	>0.9999	8	10
	DOX vs. ACN-DOX	5.950	>0.9999	8	5
	DOX vs. ACN	−1.750	>0.9999	8	10
	ACN-DOX vs. ACN	−7.700	0.7323	5	10
TWIST1	Control vs. DOX	0.008862	0.0528	10	11
	Control vs. ACN-DOX	0.006722	0.2371	10	9
	Control vs. ACN	0.006637	0.1812	10	13
	DOX vs. ACN-DOX	−0.002139	0.9237	11	9
	DOX vs. ACN	−0.002224	0.8918	11	13
	ACN-DOX vs. ACN	−8.512e−005	>0.9999	9	13
VEGFA	Control vs. DOX	0.008862	0.0528	10	11
	Control vs. ACN-DOX	0.006722	0.2371	10	9
	Control vs. ACN	0.006637	0.1812	10	13
	DOX vs. ACN-DOX	−0.002139	0.9237	11	9
	DOX vs. ACN	−0.002224	0.8918	11	13
	ACN-DOX vs. ACN	−8.512e−005	>0.9999	9	13
AMPKa2	Control vs. DOX	−3.167	>0.9999	8	12
	Control vs. ACN-DOX	−0.4643	>0.9999	8	7
	Control vs. ACN	6.341	>0.9999	8	11
	DOX vs. ACN-DOX	2.702	>0.9999	12	7
	DOX vs. ACN	9.508	0.2424	12	11
	ACN-DOX vs. ACN	6.805	>0.9999	7	11
Bcl2	Control vs. DOX	−0.1000	>0.9999	8	10
	Control vs. ACN-DOX	−1.571	>0.9999	8	7
	Control vs. ACN	8.200	0.5496	8	10
	DOX vs. ACN-DOX	−1.471	>0.9999	10	7
	DOX vs. ACN	8.300	0.4207	10	10
	ACN-DOX vs. ACN	9.771	0.3179	7	10
IL-1β	Control vs. DOX	−0.0009430	0.9610	10	12
	Control vs. ACN-DOX	0.002618	0.6143	10	8
	Control vs. ACN	0.002276	0.6422	10	12
	DOX vs. ACN-DOX	0.003561	0.3210	12	8
	DOX vs. ACN	0.003220	0.3118	12	12
	ACN-DOX vs. ACN	−0.0003415	0.9983	8	12
IL-6	Control vs. DOX	−2.125	>0.9999	8	11
	Control vs. ACN-DOX	−10.46	0.3167	8	9
	Control vs. ACN	1.775	>0.9999	8	10
	DOX vs. ACN-DOX	−8.333	0.5715	11	9
	DOX vs. ACN	3.900	>0.9999	11	10

Gene	Comparisons	Mean Diff. or Mean Rank Diff.	*P* value	*N1*	*N2*
	ACN-DOX vs. ACN	12.23	0.0995	9	10
MMP13	Control vs. DOX	−1.514	>0.9999	8	9
	Control vs. ACN-DOX	−5.125	>0.9999	8	8
	Control vs. ACN	5.708	>0.9999	8	12
	DOX vs. ACN-DOX	−3.611	>0.9999	9	8
	DOX vs. ACN	7.222	0.7815	9	12
	ACN-DOX vs. ACN	10.83	0.1700	8	12
MMP7	Control vs. DOX	−7.333	0.7378	6	8
	Control vs. ACN-DOX	−12.83	0.0694	6	6
	Control vs. ACN	−3.933	>0.9999	6	10
	DOX vs. ACN-DOX	−5.500	>0.9999	8	6
	DOX vs. ACN	3.400	>0.9999	8	10
	ACN-DOX vs. ACN	8.900	0.3016	6	10
NF-κB	Control vs. DOX	−7.333	0.7378	6	8
	Control vs. ACN-DOX	−12.83	0.0694	6	6
	Control vs. ACN	−3.933	>0.9999	6	10
	DOX vs. ACN-DOX	−5.500	>0.9999	8	6
	DOX vs. ACN	3.400	>0.9999	8	10
	ACN-DOX vs. ACN	8.900	0.3016	6	10

Relative mRNA levels were calculated using the comparative CT method, with RPL19 as housekeeping gene. Tumor tissues were collected from animals euthanized between timepoints 9 and 12. Mean Diff. or mean rank Diff. were calculated from fold change values ($2^{\wedge\Delta\Delta cq}$). Statistical analysis was conducted using one-way ANOVA followed by Tukey's multiple comparisons test for normally distributed data; otherwise, the Kruskal-Wallis test followed by Dunn's multiple comparisons test was used (#). *N1* and *N2* represent the total number of samples for each experimental group after outliers were removed by the ROUT method (Q = 10%).

Supplementary Table S3. Frequency distributions of tumor necrosis grade and mitotic index grade.

Necrotic Grade Distribution				
**Grade**	**Control**	**ACN**	**DOX**	**DOX-ACN**
1	22%	30%	27%	22%
2	11%	20%	45%	11%
3	33%	40%	0%	44%
4	33%	10%	27%	22%
N	*9*	*10*	*11*	*9*
**Mitotic Index Grade Distribution**				
**Grade**	**Control**	**ACN**	**DOX**	**DOX-ACN**
1	11%	33%	18%	33%
2	22%	11%	18%	33%
3	22%	33%	45%	22%
4	44%	22%	18%	11%
N	*9*	*9*	*11*	*9*

Tumor necrosis grade and mitotic index grade were determined by transforming data into fractions of total from all experimental groups followed by descriptive statistical analysis to determine minimum, maximum, and interquartile values. Tumor necrosis and mitotic index grades were assigned as 1 (from minimum value to < 25% percentile), 2 (from 25% percentile to < median), 3 (from median to < 75% percentile), and 4 (from 75% percentile to maximum value).

Supplementary Table S4. Metastatic area and metastatic grade distribution in lungs.

Metastatic Area (%)				
	**Control**	**ACN**	**DOX**	**DOX-ACN**
Mean	29.6	18.3	33.2	27.3
SD	6.25	8.79	10.1	9.19
SEM	1.98	2.65	3.06	3.06
**Metastatic Grade Distribution**				
**Grade**	**Control**	**ACN**	**DOX**	**DOX-ACN**
1	0%	63%	9%	22%
2	50	27%	18%	22%
3	20	9%	36%	33%
4	30	0%	36%	22%
N	*10*	*11*	*11*	*9*

Lung metastatic grades were determined by transforming area data into fractions of total from all experimental groups followed by descriptive statistical analysis to determine minimum, maximum, and interquartile values. Metastatic grades were assigned as 1 (from minimum value to < 25% percentile), 2 (from 25% percentile to < median), 3 (from median to < 75% percentile), and 4 (from 75% percentile to maximum value). *N* is the number of experimental units used for analysis.

Supplementary Table S5. Hepatic metastasis from BC tumors.

	**Metastatic Frequency And Incidence**			
	**Control**	**ACN**	**DOX**	**DOX-ACN**
	60.0% (6/10)	46.2% (6/13)	69.2% (9/13)	66.7% (6/9)
	**Metastatic Area (%)**			
	**Control**	**ACN**	**DOX**	**DOX-ACN**
Mean	0.409	0.701	0.871	1.03
SD	0.233	0.581	0.620	0.357
SEM	0.104	0.237	0.219	0.160
	**Metastatic Grade Distribution**			
**Grade**	**Control**	**ACN**	**DOX**	**DOX-ACN**
1	40%	33%	25%	0%
2	40%	16%	25%	20%
3	20%	16%	12.5%	40%
4	0%	33%	37.5%	40%

Frequency data (%) and incidence (fraction) are the number of organs with metastasis in relation to total analyzed per experimental group. Liver metastatic grades were determined by transforming metastatic area data into fractions of total from all experimental groups followed by descriptive statistical analysis to determine minimum, maximum, and interquartile values. Metastatic grades were assigned as 1 (from minimum value to < 25% percentile), 2 (from 25% percentile to < median), 3 (from median to < 75% percentile), and 4 (from 75% percentile to maximum value). Data from metastatic area was subjected to outlier removal by the ROUT method (Q = 10%).

Supplementary Table S6. Cardiac metastasis from BC tumors.

**Metastatic Frequency and Incidence**				
	**Control**	**ACN**	**DOX**	**DOX-ACN**
	50.0% (5/10)	15.4% (2/13)	53.8% (7/13)	44.4% (4/9)
**Metastatic Area (%)**				
	**Control**	**ACN**	**DOX**	**DOX-ACN**
Mean	3.40	2.62	3.24	0.908
SD	3.60	1.30	3.26	1.22
SEM	1.47	0.920	1.33	0.705
**Metastatic Grade Distribution**				
**Grade**	**Control**	**ACN**	**DOX**	**DOX-ACN**
1	16%	0%	33%	33%
2	33%	0%	0%	33%
3	16%	50%	33%	33%
4	33%	50%	33%	0%

Frequency data (%) and incidence (fraction) are the number of organs with metastasis in relation to total analyzed per experimental group. Heart metastatic grades were determined by transforming metastatic area data into fractions of total from all experimental groups followed by descriptive statistical analysis to determine minimum, maximum, and interquartile values. Metastatic grades were assigned as 1 (from minimum value to < 25% percentile), 2 (from 25% percentile to < median), 3 (from median to < 75% percentile), and 4 (from 75% percentile to maximum value). Data from metastatic area was subjected to outlier removal by the ROUT method (Q = 10%).

Supplementary Table S7. Renal metastasis from BC tumors.

**Metastatic Frequency and Incidence**				
	**Control**	**ACN**	**DOX**	**DOX-ACN**
	40% (4/10)	15.4% (2/13)	15.4% (2/13)	44.4% (4/9)
**Metastatic Area (%)**				
	**Control**	**ACN**	**DOX**	**DOX-ACN**
Mean	1.01	0.875	0.634	19.5
SD	0.635	0.597	0.258	27.3
SEM	0.366	0.422	0.182	13.6
**Metastatic Grade Distribution**				
**Grade**	**Control**	**ACN**	**DOX**	**DOX-ACN**
1	0%	50%	50%	0%
2	66%	50%	50%	25%
3	33%	0%	0%	0%
4	0%	0%	0%	75%

Frequency data (%) and incidence (fraction) are the number of organs with metastasis in relation to total analyzed per experimental group. Kidney metastatic grades were determined by transforming metastatic area data into fractions of total from all experimental groups followed by descriptive statistical analysis to determine minimum, maximum, and interquartile values. Metastatic grades were assigned as 1 (from minimum value to < 25% percentile), 2 (from 25% percentile to < median), 3 (from median to < 75% percentile), and 4 (from 75% percentile to maximum value). Data from metastatic area was subjected to outlier removal by the ROUT method (Q = 10%).

Supplementary Table S8. Splenic metastasis from BC tumors.

**Metastatic Frequency And Incidence**				
	**Control**	**ACN**	**DOX**	**DOX-ACN**
	7.0% (1/13)	0.0% (0/13)	38.5% (5/13)	40.0% (4/10)
**Metastatic Area (%)**				
	**Control**	**ACN**	**DOX**	**DOX-ACN**
Mean			0.424	2.44
SD			0.275	0.257
SEM			0.159	0.182
**Metastatic Grade Distribution**				
**Grade**	**Control**	**ACN**	**DOX**	**DOX-ACN**
1	0%	50%	50%	0%
2	66%	0%	50%	0%
3	33%	50%	0%	25%
4	0%	0%	0%	75%

Frequency data (%) and incidence (fraction) are the number of organs with metastasis in relation to total analyzed per experimental group. Data from metastatic area was subjected to outlier removal by the ROUT method (Q = 10%).

Supplementary Table S9. Primer sequences.

Primer	Forward (5′–3′)	Reverse (5′–3′)
Signal transducer and activator of transcription 3 (STAT3)	CTTGTCTACCTCTACCCCGACAT	GATCCATGTCAAACGTGAGCG
Zinc finger 1 (Snail1)	AGTTGACTACCGACCTTG	AAGGTGAACTCCACACAC
Cyclic-AMP response element-binding protein 1 (CREB)	CTTCCACTTCTGCCCTCAAG	TCCCTAAGGCAATCATGGAG
Hypoxia-inducible factor (HIF)	CGATGACACAGAAACTGAAG	GAAGGTAAAGGAGACATTGC
Phosphoinositide 3-kinase (PI3K)	GCGTGACATGTAGGCTCTCA	CAGTTTCCTTGGCTTTGCTC
Protein kinase B (Akt)-1	TGATCAAGTTCTCCTACTCAG	TCCGAGAAACAAAACACTAG
Mechanistic target of rapamycin (mTOR)	CTCAAGCGATCCAGTTGTCA	CAAAGAAGGGCTGAACTTGC
Cluster of differentiation 44 (Cd44)	GAATTAGCTGGACACTCAAG	CACCTTCTCCTACTATTGACC
Transforming growth factor beta (TGFβ1)	GGATACCAACTATTGCTTCAG	TGTCCAGGCTCCAAATATAG
Vimentin (Vim)	GAACCTGAGAGAAACTAACC	GATGCTGAGAAGTCTCATTG
Tight junction protein 1 (Tjp1)	CTGATAGAAAGGTCTAAAGGC	TGAAATGTCATCTCTTTCCG
I-kappa-B kinase beta (IKKβ)	GCTGCTTCAGGCAATCC	TGTCTGGGCTTCCACTCACG
Nuclear factor kappa-light-chain-enhancer of activated B cells (NF-κB)	GGATGGTGAGGTCACTCT	TCCTGAACTCCAGCACTCTCTTC
Sirtuin 1 (SIRT1)	TTGTGAAGCTGTTCGTGGAG	GGCGTGGAGGTTTTTCAGTA
Response gene to complement (Rgcc)32	CCTTAGCAAGTATGTGAAGC	CCCAATGGCTAATGTTATAGTG
E-cadherin (Cdh1)	CATGTTCACTGTCAATAGGG	GTGTATGTAGGGTAACTCTCTC
Centromere protein F (Cenpf)	GACTTACCCAGGAGTTACAG	CTTCATTTCCTCACTGCTTC
Protein kinase AMP-activated catalytic subunit alpha 2 (Prkaa2)	AGACTATCTCAACCGTTCTG	CCATTCATGTTCTCGTATGTC
Twist family BHLH transcription factor (Twist1)	GAGACCTAGATGTCATTGTTTC	GAATTTGGTCTCTGCTCTTC
Vascular endothelial growth factor A (VEGFA)	TAGAGTACATCTTCAAGCCG	TCTTTCTTTGGTCTGCATTC
Activated protein kinase alpha 2 (AMPKa2)	TGGCTGCCTTCTTATGCTTT	GCTTTGAAACGGCTTCTCAC
B-cell lymphoma 2 (Bcl2)	AGGAGCTGCCTACAAGA	GCATTTTCCCACTGTCT
Interleukin (IL)-1β	TCGCTCAGGGTCACAAGAAA	CATCAGAGGCAAGGAGGAAAAC
Interleukin (IL)-6	AGTTGCCTTCTTTGGGACTGA	TCCACGATTTCCCAGAGAAC
Matrix metallopeptidase (MMP)13	CAGTTGACAGGCTCCGAGAA	TTCACCCACATCAGGCACTC
Matrix metallopeptidase (MMP)7	TGAATTTGGCCACTCTCTGGGTCT	TCTGAATGCCTGCAATGTCGTCCT
Ribosomal protein L19 (RPL19)	GAAGGTCAAAGGGAATGTGTTC	CCTTGTCTGCCTTCAGCTTGT
β-Actin	TGTTACCAACTGGGACGACA	GGGGTGTTG. AAGGTCTCAAA
